# Polyaniline nano-material backed lens antenna for X-band LEO satellite transceivers

**DOI:** 10.1038/s41598-025-14270-y

**Published:** 2025-08-17

**Authors:** H. Abuklill, Khalid F. A. Hussein, A. A. Shaalan, Mohamed E. Nasr, A. M. Elshaer

**Affiliations:** 1 Department of Communications and Electronics, Alexandria Higher Institute for Engineering and Technology, Alexandria, Egypt; 2 Department of Microwave Engineering, Electronics Research Institute (ERI), Cairo, Egypt; 3 Electronics and Communication Department, Faculty of Engineering, Zagazig University, Zagazig, Egypt; 4 Department of Electronics and Electrical Communications Engineering, Faculty of Engineering, Tanta University, Tanta, Egypt; 5 College of Artificial Intelligence, Arab Academy for Science, Technology, and Maritime Transport, P.O. B 1029, Alamein, Egypt

**Keywords:** Nano-materials in antennas, Polyaniline (PANI) absorber, Isoflux antenna, Dielectric lens antenna, X-band satellite communication, Low earth orbit (LEO), Conical beam pattern, Engineering, Design, synthesis and processing

## Abstract

This paper presents the design and implementation of an open-ended waveguide lens antenna engineered to generate a conical-shaped radiation pattern for the X-band fully-duplex communication subsystem of a low Earth orbit (LEO) satellite dedicated to Earth remote sensing. The antenna is designed to maintain reliable links with ground stations at a minimum satellite elevation angle of 10°, corresponding to ± 63° off-nadir, for a near-circular orbit at an altitude of 700 km. Operating within the 9.75–10.25 GHz band, the antenna provides broad coverage over a working sector of approximately 126° × 126° in azimuth and elevation, with peak radiation directed at ± 63° from nadir. It achieves a minimum gain of 5 dBic in these directions and employs right-hand circular polarization (RHCP) for downlink and left-hand circular polarization (LHCP) for uplink. The axial ratio remains below 3 dB across the working sector at the center frequency of 10 GHz, while the input reflection coefficient stays better than − 10 dB over a wide impedance matching bandwidth of 8.75–11.25 GHz. The 3-dB axial ratio bandwidth spans from 9.75 to 10.25 GHz. To further enhance performance particularly circular polarization purity, gain, and axial ratio bandwidth a metallic circular backing plate coated with a polyaniline (PANI) nano-material absorber is integrated between the dielectric lens and the reflector disc. The PANI layer, characterized by tunable dielectric properties and intrinsic microwave loss, improves impedance matching at the lens–waveguide interface and effectively suppresses surface currents and backward radiation. Material characterization via X-ray diffraction (XRD) and scanning electron microscopy (SEM) confirms the semi-crystalline structure and micro-porous morphology of the synthesized PANI, which contribute to enhanced electromagnetic absorption. As a result, the axial ratio at 10 GHz is reduced from 1.0 dB to 0.05 dB, the gain at ± 63° is increased from 4.1 dBic to 6.0 dBic, and the 3-dB axial ratio bandwidth is expanded from 400 MHz to 500 MHz. These findings demonstrate the potential of integrating functional PANI nano-materials into high-performance antenna architectures for advanced satellite communication and Earth observation applications.

## Introduction

The increasing demand for high-capacity, reliable satellite communication systems for Earth observation, remote sensing, and global connectivity has driven extensive research into the design of efficient antenna subsystems for low Earth orbit (LEO) satellites^1–3^. Among the various antenna types, open-ended circular waveguide antennas have gained attention due to their structural simplicity, mechanical robustness, and compatibility with circular polarization (CP) schemes—critical for mitigating polarization mismatch and Faraday rotation effects in satellite links^4,5^.

For modern LEO satellite missions operating in the X-band (8–12 GHz), ensuring consistent ground coverage with minimal onboard complexity is a priority. Isoflux antennas, which maintain nearly constant flux density across the Earth’s surface by producing a conical radiation pattern, are especially advantageous for these applications^6–8^. A typical isoflux profile requires an antenna to generate a main lobe at large off-nadir angles (e.g., ± 63°), enabling reliable link budgets even at low elevation angles of ~ 10° from the ground station perspective^9,10^.

Traditionally, various reflector and array-based approaches have been adopted to realize conical beam shapes^11,12^. However, such systems may suffer from increased weight, complexity, and limited scalability. Lens-based antennas, in contrast, offer compactness and natural beam shaping through geometric optics principles^13^. Open-ended waveguide antennas augmented with dielectric lenses provide a viable approach to achieve desired beam control while preserving mechanical simplicity^14–16^.

A number of studies have investigated the use of dielectric lenses in conjunction with circular waveguides for beam shaping. For instance, Tan et al.^14^ proposed a Teflon-based lens mounted on a circular waveguide to obtain directional CP radiation. Similarly, in^15^, Liu et al. explored a cone-shaped dielectric lens for wide-angle coverage in satellite communications, highlighting the role of dielectric permittivity and lens profile in controlling the beamwidth and sidelobe levels.

In addition to beam shaping, polarization purity and backward radiation suppression remain critical for effective duplex communication. To address these issues, metamaterial absorbers and lossy dielectric materials have been used to damp spurious modes and surface currents^17,18^. Among such materials, conducting polymers like polyaniline (PANI) have shown promise due to their tunable electromagnetic properties, lightweight nature, and ease of fabrication^19–21^. When applied as thin absorbing layers, PANI-based materials can enhance antenna performance by reducing axial ratio, improving gain stability, and mitigating multipath effects^22^.

Recent studies, such as Kim et al., demonstrated the use of carbon-based absorbers in suppressing sidelobes in waveguide-fed lens antennas, while Elshazly et al. reported performance enhancements using PANI composites in microwave absorbers^23,24^. The present work builds upon these developments by integrating a micro-porous PANI nano-material layer between the lens and a metallic reflector disc to suppress backward leakage and improve the forward radiation characteristics of a conical-beam lens antenna for LEO satellite systems.

Recent literature further supports the relevance and applicability of the proposed design. For instance, the study in^25^ demonstrates a dielectric circular polarizer integrated into a horn antenna, achieving wideband circular polarization with excellent impedance matching and axial ratio performance. This highlights the growing interest in polarization control using dielectric or coaxial polarizers, which is closely aligned with the methodology adopted in our work. Similarly, the work in^26^ presents a dual circularly polarized CubeSat antenna designed for LEO missions, emphasizing broadband performance, polarization purity, and full spacecraft qualification. Such compact, mission-ready circularly polarized antennas underscore the practical viability of our design approach for small satellite platforms. In^27^, a quadrifilar helix antenna is reported with isoflux radiation characteristics and circular polarization—structurally and conceptually aligned with the intended radiation pattern of our antenna. This reinforces the relevance of wide-beam CP antennas for ensuring uniform Earth coverage in satellite communications. Moreover, the study in^28^ introduces a circularly polarized omnidirectional slot-waveguide antenna for TT&C applications, illustrating the practical trade-offs between gain, polarization, and compactness. These recent developments collectively validate the technical foundation and practical direction of our antenna design within the evolving landscape of satellite communication systems.

The key contributions of this paper include: (i) *Dual-port circular polarization excitation* using a broadband orthomode transducer (OMT) feeding mechanism with measured isolation better than 20 dB across the X-band. This configuration ensures efficient Left-Hand Circular Polarization (LHCP) for transmission and Right-Hand Circular Polarization (RHCP) for reception—meeting the stringent demands of full-duplex LEO satellite terminals. (ii) *The integration of a top-cut dielectric lens* to achieve a conical beam covering ± 63° from nadir. (iii) *Integration of a DBSA-doped polyaniline (PANI) nano-material absorber* between the dielectric lens and metallic reflector in an open-ended circular waveguide antenna. While prior lens antennas have used dielectric matching or traditional absorbing layers, the incorporation of a conducting polymer-based nano-material is novel and experimentally validated in the present work. (iv) *Enhanced radiation performance due to PANI absorber placement*. Unlike conventional absorbers placed externally or behind the reflector, we position the absorber directly between the lens base and the reflector. This configuration simultaneously suppresses surface waves, reduces sidelobes, and improves gain uniformity and axial ratio bandwidth without significantly increasing antenna size.

The time-domain solver in CST Studio Suite is employed for electromagnetic simulation. This simulator is based on the Finite Integration Technique (FIT). This method solves Maxwell’s equations in the time domain using a hexahedral mesh and is well-suited for broadband electromagnetic analysis. Using this simulator, a detailed parametric study examining probe position, reflector dimensions, lens geometry, and absorber properties, followed by performance validation through full-wave simulations.

The proposed antenna achieves a minimum gain of 5.4 dBic at the targeted off-nadir angles, a 3-dB axial ratio bandwidth of 500 MHz (9.75–10.25 GHz), and an impedance matching bandwidth spanning from 8.75 to 11.25 GHz. These results demonstrate the effectiveness of combining lens-based beam shaping with nano-material-assisted absorption in realizing high-performance conical-beam antennas for satellite applications.

The remainder of this paper is structured as follows. Section “Antenna design” presents the detailed design methodology of the proposed open-ended waveguide lens antenna, including its dual-port excitation scheme, dielectric lens geometry, and reflector configuration. Section “Preparation of PANI nano-material” describes the synthesis and characterization of the PANI nano-material absorber, highlighting its structural, morphological, and electromagnetic properties. Section “Antenna fabrication and measurement” outlines the fabrication process of the antenna prototype and the experimental setup used for performance measurements in an anechoic environment. Section “Results and discussions” discusses the simulated and measured results, including impedance matching, gain patterns, axial ratio performance, and the comparative impact of integrating the PANI absorber. Finally, Sect. “Comparison with related works” summarizes the key findings and contributions of the work, emphasizing the effectiveness of nano-material integration in enhancing the performance of advanced conical-beam antennas for LEO satellite communication systems.

## Antenna design

The proposed antenna is designed as an open-ended circular waveguide lens antenna operating in the $9.75{-}10.25{\text{ GHz}}$ ($500{\text{ MHz}}$ bandwidth) for a fully-duplex communication subsystem on a LEO satellite. The primary objective of the antenna is to generate a broad conical-shaped radiation pattern with CP, enabling reliable data transmission and reception with Earth-based ground stations at low elevation angles $( \ge 10^\circ$), corresponding to radiation maxima at approximately $\pm 63^\circ$ from nadir. To meet these specifications, the antenna integrates several key structural and electromagnetic design features.

**Fig. 1:**
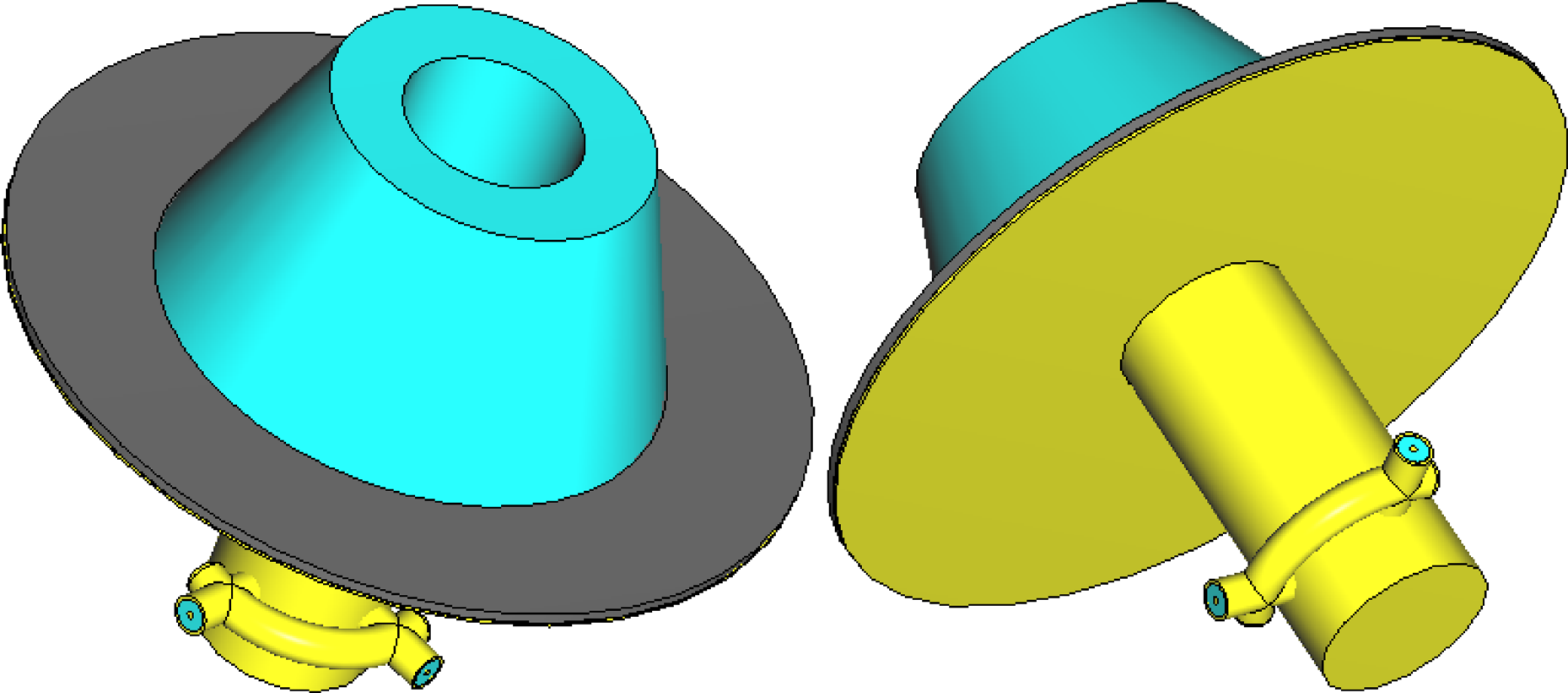
Three-dimensional views of the proposed antenna illustrating both the top and bottom geometries. Figure generated by the authors using Microsoft Word (Office 2010) and Microsoft Paint (Windows 10).

**Fig. 2:**
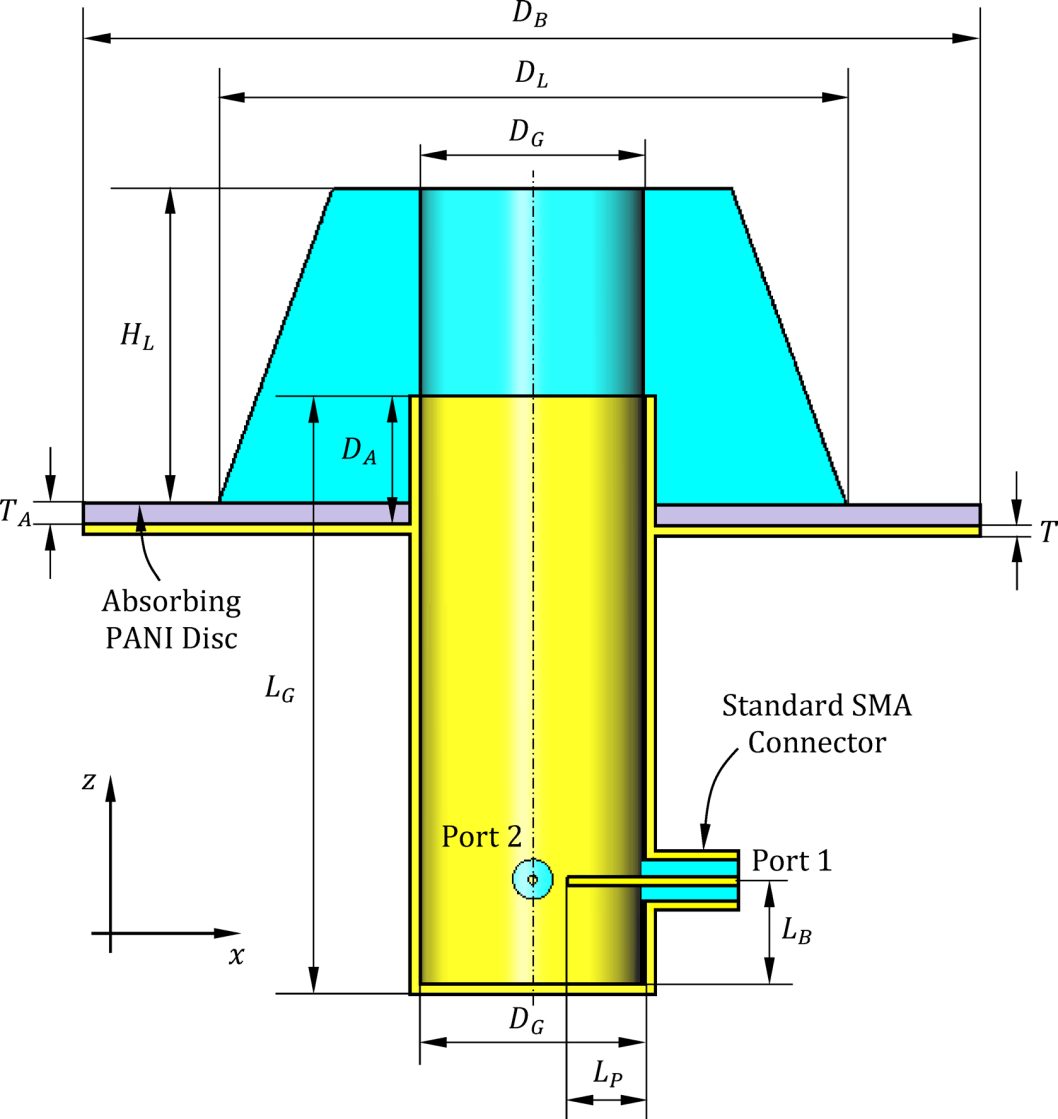
Longitudinal ($zx$) section of the proposed lens-terminated open-ended waveguide antenna.

Figures 1 and 2 illustrate the structure and geoetry of the proposed lens antenna. The radiating element consists of a circular waveguide terminated with a dielectric lens and backed by a circular metallic disc. The waveguide operates in its dominant $TE_{11}$ mode and is excited via two orthogonally positioned coaxial probes that penetrate its sidewall. These probes terminate a $90^\circ$ arc-shaped coaxial feed line aligned with the waveguide perimeter near the closed end. Each probe is connected to an SMA port (Port 1 or Port 2), and their orthogonal geometry, combined with a $90^\circ$ phase difference at the center frequency ($10{\text{ GHz}}$), ensures circular polarization generation. Excitation through Port 1 results in right-hand circular polarization (RHCP) for transmission, while Port 2 excitation yields left-hand circular polarization (LHCP) for reception, thus enabling duplex operation using the same aperture with polarization diversity.-

To achieve the desired broad conical beam with a main lobe directed at $\pm 63^\circ$ from nadir, a top-cut conical dielectric lens is mounted concentrically above the waveguide’s open end. The lens collimates the radiated field into a conical pattern that covers a working sector of approximately $126^\circ \times 126^\circ$ in azimuth and elevation. The backing circular disc beneath the lens serves as a reflector that enhances forward radiation while suppressing backward leakage.

To further enhance polarization purity, bandwidth, and gain performance, a thin layer of PANI nano-material is coated on the backing plate facing the dielectric lens. This electromagnetic absorber mitigates surface wave reflections and fine-tunes the impedance environment of the lens-waveguide junction. Experimental integration of the PANI layer improves the axial ratio from 1.0 dB to 0.05 dB at the beam maxima (± 63°), increases gain from $4.1{\text{ dBic}}$ to $5.4{\text{ dBic}}$, and broadens the $3 - {\text{dB}}$ axial ratio bandwidth from $400{\text{ MHz}}$ to $500{\text{ MHz}}$.

Overall, the antenna design effectively satisfies the key system requirements of X-band operation, dual CP, wide beam coverage at low elevation angles, and enhanced performance via material-assisted electromagnetic conditioning, making it suitable for Earth remote sensing LEO satellite missions.

### Electromagnetic simulator used for antenna design development

The CST Studio Suite is employed to develop the final design of the proposed Isoflux antenna. The CST employs the *Finite Integration Technique (FIT)* as the core numerical foundation for its transient (time-domain) simulations. FIT discretizes Maxwell’s equations in their integral form and supports structured as well as unstructured grids, providing enhanced flexibility over traditional FDTD methods. Notably, FIT retains a generalized matrix–vector formalism and is capable of accurately modeling complex 3D structures, curved geometries, and inhomogeneous materials through advanced techniques such as the Perfect Boundary Approximation (PBA) and the Thin Sheet Technique (TST).

Although FIT and FDTD share conceptual similarities, particularly in their explicit time-stepping approaches on Cartesian grids, FIT is more general and serves as a superset of FDTD. The distinction between the two is well documented in CST’s official documentation and technical literature. Therefore, we have carefully revised the manuscript to correctly state that the transient solver in CST Studio Suite is based on the Finite Integration Technique (FIT) rather than FDTD.

### Design stages of the IsoFlux antenna

This section outlines the systematic development of the proposed isoflux antenna through a sequence of incremental design stages, each addressing a critical functional requirement for spaceborne wide-beam circularly polarized radiation. The process begins with the design of a dual-port polarizer and waveguide feeding structure to enable LHCP transmission and RHCP reception. It then proceeds with the configuration of the open-ended circular waveguide and an external metallic reflecting disc, which together form the basic radiating structure. To achieve the broad conical beam required for uniform Earth coverage from LEO, a dielectric lens is introduced above the waveguide aperture. The performance is further refined by inserting a nano-material absorbing disc made of PANI between the lens base and the reflector to suppress backlobes and enhance forward radiation. Finally, performance results are presented to demonstrate the effectiveness and cumulative improvements of each design stage toward meeting the stringent requirements of X-band LEO satellite communication systems.

#### Design of the polarizer and feeding ports

In conventional designs, a circular waveguide is commonly excited using a single probe that is an extension of the inner conductor of a coaxial line, such as an SMA connector. The probe is inserted into the waveguide wall, typically oriented perpendicular to the waveguide axis, and positioned approximately a quarter wavelength $\lambda_{g} /4$) from the shorted (closed) end to achieve efficient coupling. The probe length is usually optimized to resonate near a quarter wavelength for maximum energy transfer. This configuration predominantly excites the fundamental $TE_{11}$ mode due to its field distribution aligning with the probe’s orientation, enabling effective mode excitation and impedance matching.

**Fig. 3:**
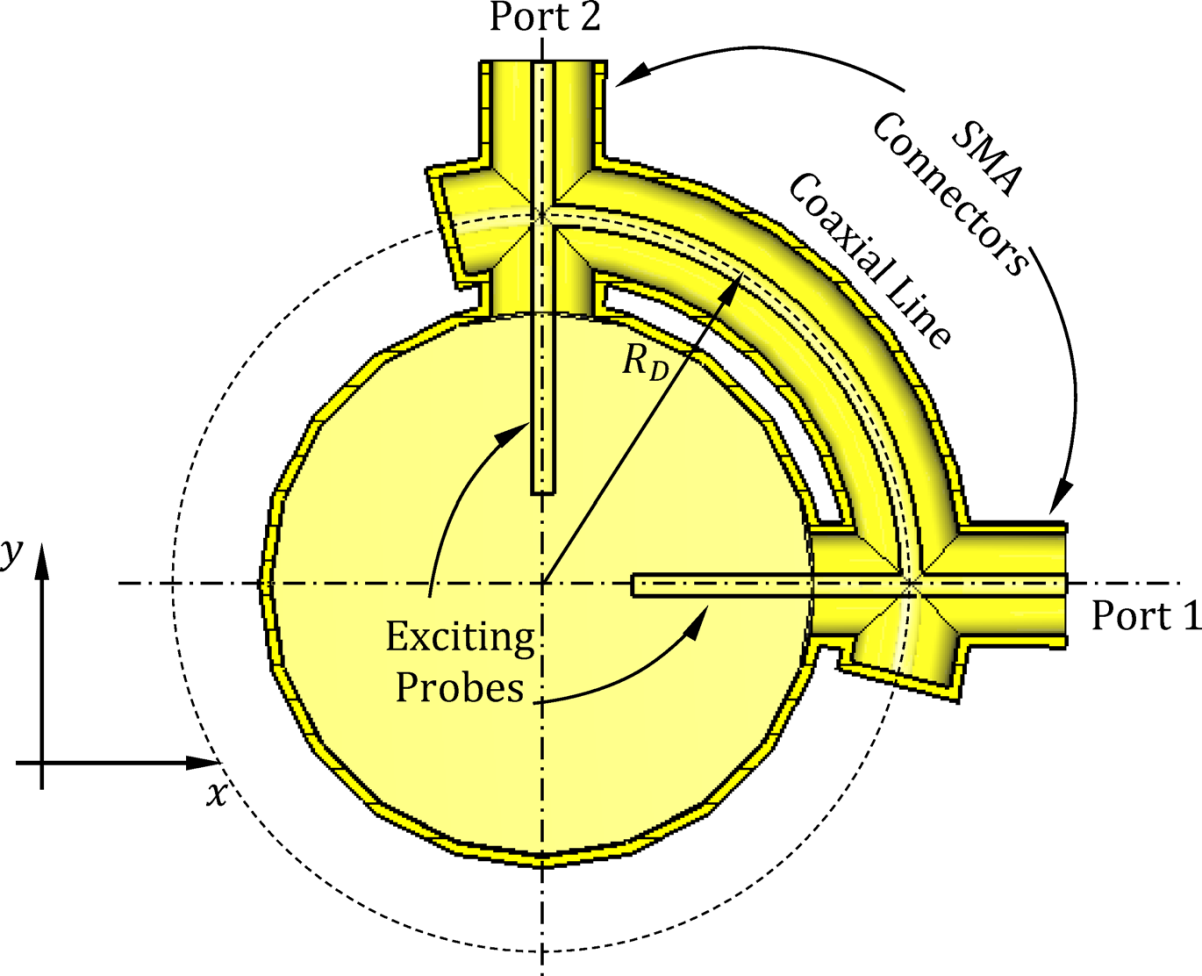
Cross-sectional view of the polarizer structure in the plane of the curved coaxial line. The figure illustrates the quarter-circular coaxial section connecting ports 1 and 2 via SMA connectors, with orthogonally oriented inner probes extending radially into the circular waveguide. The 90° arc of the coaxial line introduces the required phase shift between the probes, enabling circular polarization.

For exciting the proposed circularly polarized antenna, the polarizer is designed as a quarter-circlular Sect. (90° arc) of a coaxial transmission line, as shown in Fig. 3, with standard SMA connectors mounted at each end, designated as port 1 and port 2. The outer conductors of the SMA connectors are securely welded to the outer conductor of the arc-shaped coaxial line, ensuring continuous electrical shielding. The inner conductors of the SMA connectors are connected to the inner conductor of the coaxial line and extend radially into the circular waveguide, forming a pair of orthogonal probes. These probes serve as the excitation elements for the waveguide and are oriented 90 degrees apart in the azimuthal plane. The arc-shaped geometry of the coaxial line introduces a 90° phase difference between the signals at the two ports, thereby generating a CP wave within the waveguide. This structure enables the antenna to support either LHCP or RHCP, depending on the feeding port.

**Fig. 4:**
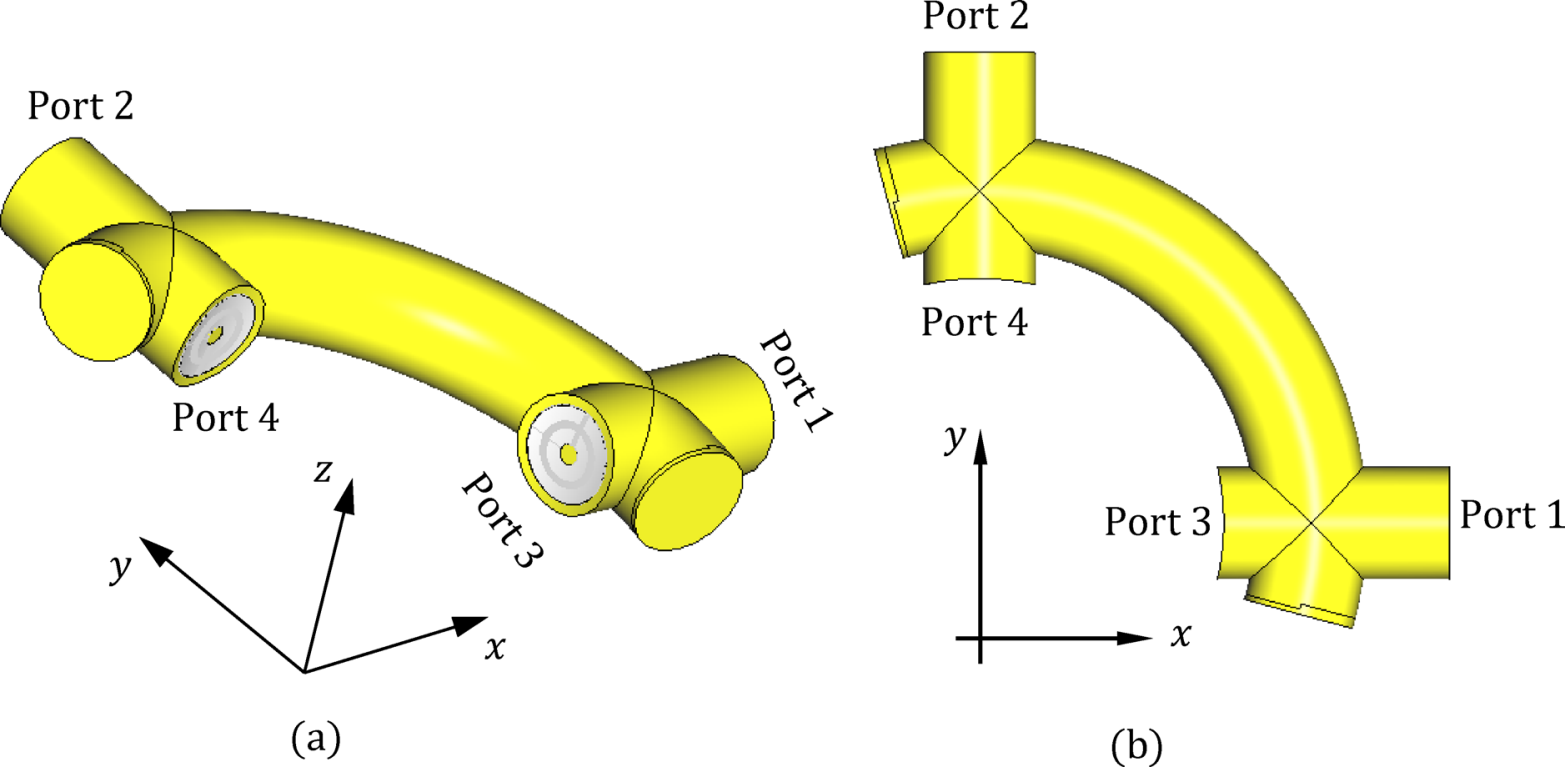
Geometry of the designed quarter-wavelength coaxial polarizer modeled in CST Studio Suite: (**a**) 3D perspective view showing the four-port configuration and curved transmission line section; (**b**) top view highlighting the quarter-circular coaxial line connecting ports 1 and 2 with a total arc length of $\lambda_{g} + \frac{1}{4}\lambda_{g}$ at 10 GHz.

To evaluate the polarizer’s capability to generate the required circular polarization through phase quadrature, spatial orthogonality, and amplitude balance between the excitation field components, the quarter-wavelength polarizer is modeled independently in CST Studio Suite as a four-port coaxial network, as illustrated in Fig. 4. This standalone simulation allows for isolated examination of the polarizer’s performance, separate from the rest of the antenna structure. Ports 1 and 2 serve as the excitation ports, while ports 3 and 4 are designated as the output ports. The structure is designed to operate at 10 GHz as the center frequency, where the guided wavelength in the coaxial line is calculated as, $\lambda_{g} = 21.21\;{\text{mm}}$, based on the dielectric-filled geometry. To achieve the desired polarization conversion, the polarizer includes a quarter-circular coaxial transmission line section between ports 1 and 2, with a total arc length of 26.5 mm. This length corresponds to $\lambda_{g} + \frac{1}{4}\lambda_{g}$​, ensuring an appropriate phase transformation for circular polarization. The corresponding radius of curvature of the quarter-circle is therefore 16.87 mm. When the structure is excited at port 1 with port 2 short-circuited, the signal arriving at the output ports exhibits a 90° phase difference: the signal at port 3 leads the signal at port 4 by 90°. Conversely, when excited at port 2 with port 1 short-circuited, the output at port 3 lags behind the output at port 4 by 90°. This phase behavior confirms the polarizer’s functionality in producing orthogonal circular polarizations depending on the excitation port.

**Fig. 5:**
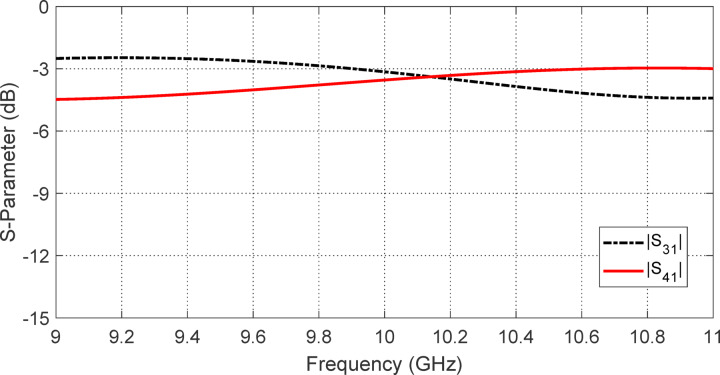
Simulated S-parameters of the four-port coaxial polarizer shown in Fig. 4. When the structure is excited at port 1, the transmission coefficients $S_{31}$ and $S_{41}$ exhibit nearly equal magnitudes around − 3 dB over a wide frequency band centered at 10 GHz, confirming even power splitting between ports 3 and 4. Similar behavior is observed for $S_{42}$ and $S_{32}$​ when the excitation is applied at port 2.

As illustrated in Fig. 5, the simulated S-parameters $S_{31}$ and $S_{41}$ of the four-port coaxial polarizer (depicted in Fig. 4) exhibit nearly identical magnitudes, both approaching –3 dB across a broad frequency range centered around 10 GHz. This behavior is observed when the polarizer is excited at port 1, with port 2 terminated. The –3 dB level indicates that the input power is evenly split between output ports 3 and 4, confirming that the structure functions effectively as a half-power divider. A similar power division is observed when the excitation is applied to port 2 instead: the corresponding S-parameters $S_{42}$ and $S_{32}$​ also maintain close proximity to ${-}3\;{\text{dB}}$ over the same frequency range, further validating the symmetric and broadband operation of the polarizer.

**Fig. 6:**
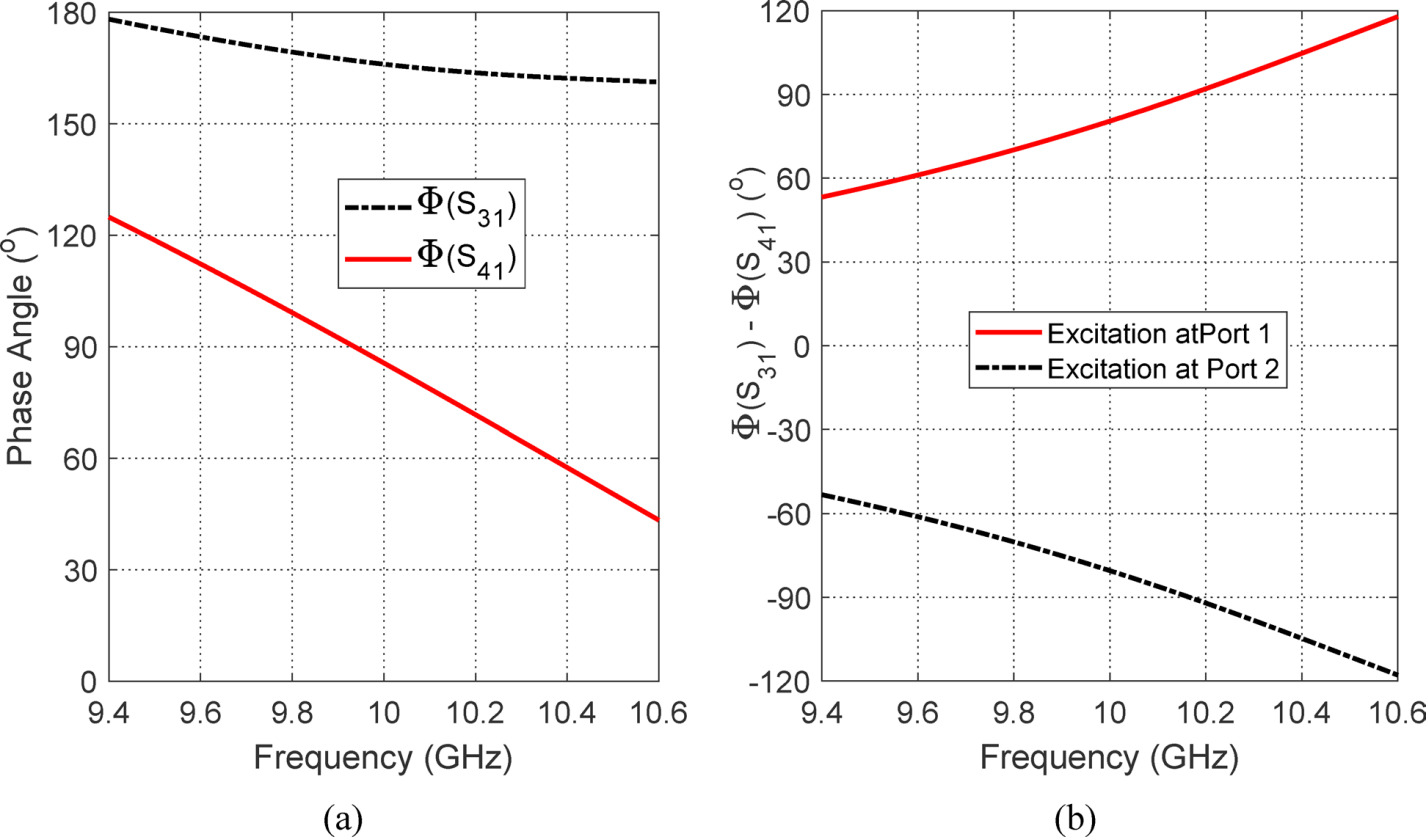
Phase behavior of the four-port coaxial polarizer when excited at port 1: (**a**) Variation of the absolute phases of $S_{31}$ and $S_{41}$​ with frequency around 10 GHz; (**b**) Phase difference ${\Phi }(S_{41} ) - {\Phi }(S_{41} )$ over the same frequency range. The consistent 90° phase lead of $S_{31}$​ relative to $S_{41}$ confirms the polarizer’s ability to generate circular polarization with proper quadrature phase shift across the operational bandwidth.

Figure 6 provides insight into the phase behavior of the four-port coaxial polarizer when excited at port 1. In Fig. 6a, the absolute phases of the transmission coefficients $S_{31}$ and $S_{41}$​ are plotted as functions of frequency in the vicinity of 10 GHz. Figure 6b presents the corresponding phase difference between $S_{31}$ and $S_{41}$​ over the same frequency range. As observed, $S_{31}$ consistently leads $S_{41}$​ by approximately 90°, with minimal deviation across the operational bandwidth. This stable 90° phase difference between the signals at ports 3 and 4 is a key requirement for generating circular polarization. It confirms that the polarizer is correctly designed to impose the intended quadrature phase shift between the two output ports. When combined with equal magnitude outputs (as demonstrated in Fig. 5), this phase relationship enables the synthesis of circularly polarized waves with a low axial ratio, validating the effectiveness of the polarizer across the desired frequency band.

#### Design of open-ended waveguide and reflecting disc

**Fig. 7:**
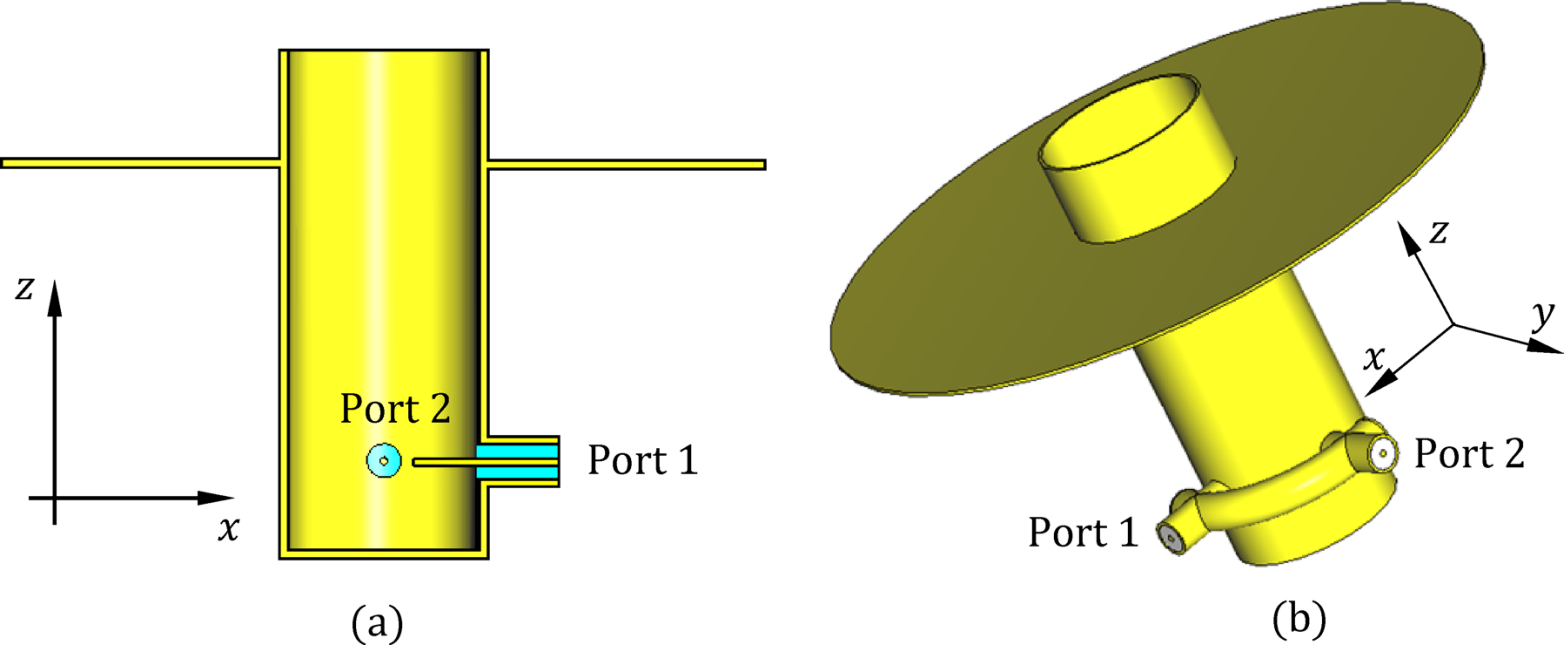
The open-ended circular waveguide antenna with dual-port polarizer excitation. The probes of length $L_{P}$ are positioned at a distance $L_{B}$​ from the closed end. The metallic reflector disc is welded to the outer wall of the waveguide at a distance $D_{A}$​ ​ from the open end. (**a**) Longitudinal section. (**b**) Three-dimensional view. Figure generated by the authors using Microsoft Word (Office 2010) and Microsoft Paint (Windows 10).

At this initial stage, the antenna primarily consists of an open-ended circular waveguide that serves as the main radiating aperture. The waveguide is excited by a dual-port polarizer, where two probes, each of appropriate length $L_{P}$​, xtend into the waveguide at a precisely selected distance, denoted as $L_{B}$​, from the waveguide’s closed end (as illustrated in Fig. 7). This careful placement ensures the optimal excitation of orthogonal waveguide modes necessary to achieve the desired circular polarization while maintaining proper impedance matching. A key structural element introduced at this stage is a metallic circular reflector disc, which is attached externally to the waveguide. This disc is securely welded to the outer wall of the circular waveguide at a distance $D_{A}$​ from the open end, measured along the waveguide’s longitudinal axis. Acting as a passive electromagnetic component, the external reflector disc modifies the boundary conditions near the waveguide aperture, thereby influencing the antenna’s radiation characteristics. By positioning the reflector disc outside and in close proximity to the waveguide aperture, the design effectively shapes the radiated wavefront and suppresses unwanted modes or spurious radiation emanating from the open end. This configuration also impacts the antenna’s input impedance, radiation pattern, and polarization purity, resulting in improved gain and reduced sidelobes, even before the addition of further components such as the dielectric lens or absorber materials.

This design stage, which includes the waveguide, dual-port polarizer, and the external reflector disc, establishes the foundational electromagnetic environment. It prepares the antenna for subsequent performance enhancements through the integration of a dielectric lens at the waveguide aperture to increase directivity and gain, and the application of a polyaniline-based absorber behind the antenna to reduce backlobes and improve overall antenna efficiency.

#### Design of dielectric lens for conical beam generation

Before the addition of the dielectric lens, the antenna’s radiation pattern, formed by the open-ended circular waveguide with the external reflector disc, exhibits a relatively wide beam but lacks precise control to produce the desired conical beam shape and directivity. The main lobe is broad and somewhat diffuse, limiting the antenna’s ability to concentrate energy in a specific angular sector required for the target application. To achieve the desired broad conical beam with a main lobe directed at approximately $\pm 63^\circ$ from nadir, a top-cut conical dielectric lens is mounted concentrically above the waveguide’s open end. This lens is perforated with a central hole matching the waveguide diameter, enabling it to be precisely mounted onto the circular reflector disc welded to the outer wall of the waveguide. The dielectric lens functions to collimate and shape the radiated fields into a well-defined conical beam pattern, covering a wide working sector of approximately $126^\circ \times 126^\circ$ in both azimuth and elevation. This carefully shaped beam provides the uniform angular coverage necessary for an isoflux antenna designed for LEO satellite applications at an altitude of 700 km. Such a beam pattern ensures that the antenna maintains consistent signal strength across the satellite’s Earth footprint, which is essential for reliable communications.

**Fig. 8:**
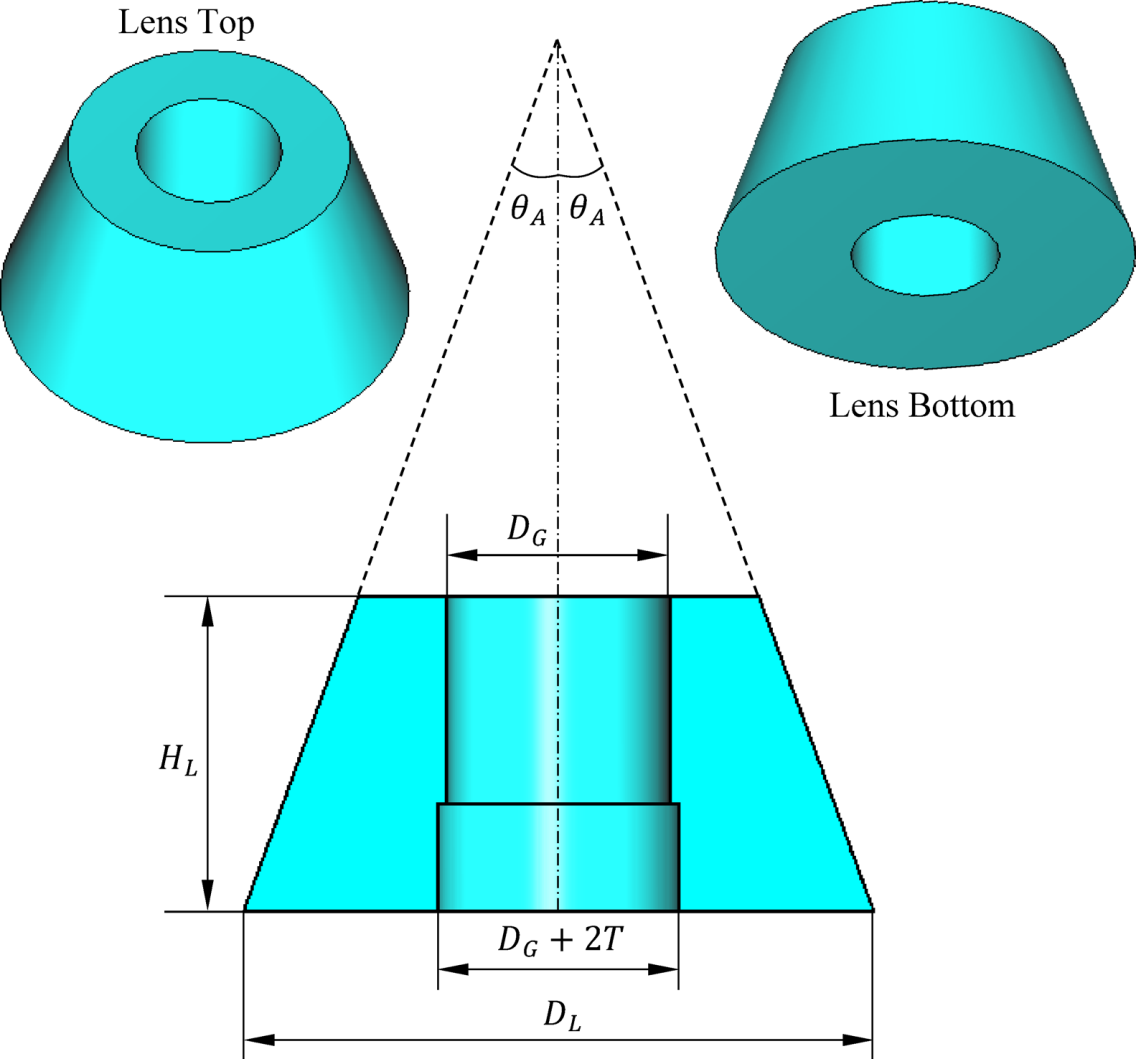
Geometry of the lens placed at the open end of the circular waveguide.

Constructed from Teflon with a dielectric constant of 2, the lens material offers low electromagnetic loss and suitable dielectric properties at the operating X-band frequencies. The geometry of the proposed lens is shown in Fig. 8. Its dimensions, including the half-apex angle ($\theta_{A}$​), base diameter ($D_{L}$​), and height ($H_{L}$​), are optimized to achieve the required beam shape and directivity for the isoflux pattern described previously. Beneath the lens, the backing circular reflector disc serves as a reflector that enhances forward radiation by redirecting backward-leaked energy. This reduces backlobes and improves antenna gain, further refining the overall radiation efficiency and pattern control. Together, the dielectric lens and the backing reflector disc transform the initial broad and less controlled radiation of the waveguide into a precisely shaped conical beam suitable for the demanding requirements of LEO satellite communication systems.

#### The absorbing disc of PANI nano-material

In the final design stage, the antenna is enhanced by incorporating an absorbing disc made of PANI nano-material as shown in Fig. 1, which is inserted between the base of the dielectric lens and the metallic circular reflector disc welded to the waveguide’s outer wall. This strategic placement allows the PANI absorbing disc to effectively suppress unwanted backlobes and reduce electromagnetic energy leakage in the backward direction. By absorbing scattered and reflected waves that would otherwise propagate backward or interfere with the antenna structure, the absorber improves the antenna’s radiation efficiency and pattern purity. The PANI nano-material is characterized by its tailored electromagnetic properties, including high complex permittivity and permeability, providing strong broadband microwave absorption at the antenna’s operational X-band frequencies. Its thin, conformal form factor allows integration without increasing the antenna’s overall size or weight. Positioned between the lens and reflector, the absorbing disc enhances forward radiation by minimizing backward leakage, reducing sidelobes, and suppressing spurious modes. This leads to improved gain, a cleaner beam pattern, and better polarization purity.

Together with the dielectric lens and the metallic reflector disc, the PANI absorbing disc completes the antenna’s final configuration, delivering an optimized radiation pattern and efficiency suited for demanding LEO satellite communication applications.

#### Optimum dimensions of the isoflux antenna

The optimal dimensions of the proposed isoflux antenna, determined to effectively achieve the aforementioned design objectives, are summarized in Table 1. These values were derived through an extensive parametric study conducted via full-wave simulations, aiming to maximize antenna performance in terms of impedance matching, axial ratio, gain pattern, and beam shape. Section “Design stages of the isoflux antenna” presents selected results and discussions as representative examples from the comprehensive parametric analysis carried out during the antenna design process.

**Table 1:** Optimum dimensions of the proposed isoflux antenna.

Parameter	${L}_{G}$	${D}_{G}$	${D}_{B}$	${L}_{B}$	${R}_{D}$	${L}_{P}$	${D}_{A}$	${D}_{L}$	$H_{L}$	$\theta_{A}$	$T_{A}$
Value (mm)	$65$	$23.83$	$100$	$11$	$16.87$	$8$	$12$	$70$	$35$	$20^\circ$	$1.5$

#### Results showing performance improvement with the design stages

This section presents a systematic investigation into the enhancement of the antenna’s performance through a sequence of progressive design stages. Beginning with a baseline configuration that excludes any lens or absorber, the study explores the incremental effects of introducing a dielectric lens, followed by the combined implementation of both the lens and a microwave absorber layer. By evaluating key performance metrics such as impedance matching (|S_11_|), axial ratio, and gain pattern across these three configurations, the section demonstrates how each design element contributes to the optimization of the antenna’s radiation characteristics. The comparative analysis, illustrated in Figs. 6, 7 and 8, highlights the advantages of the integrated lens and absorber approach in achieving improved impedance bandwidth, enhanced polarization purity, and the desired conical beam shape essential for uniform Earth coverage in LEO satellite applications.

**Fig. 9:**
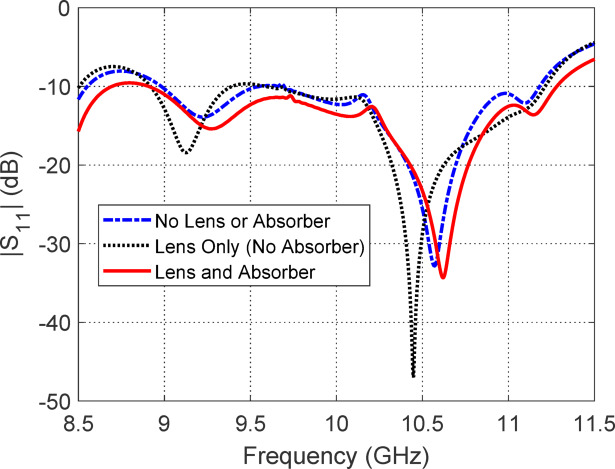
Comparison of the frequency responses of $\left| {S_{11} } \right|$ for three configurations: (i) without lens or absorber, (ii) with lens only (no absorber), and (iii) with both lens and absorber. The configuration incorporating both the lens and absorber demonstrates the best impedance matching performance over the widest frequency range.

Figure 9 illustrates the comparative frequency responses of the reflection coefficient |S_11_| for three distinct antenna configurations: (i) without lens or absorber, (ii) with a lens only (no absorber), and (iii) with both lens and absorber. In case (i), the antenna exhibits poor impedance matching across the frequency band due to the absence of any front-end electromagnetic structure to condition the incident wavefront, leading to significant reflections at the aperture. In case (ii), the addition of a dielectric lens improves the wavefront collimation and reduces the impedance mismatch between the antenna and free space, resulting in moderate improvement in |S_11_| performance. However, residual reflections still persist due to the partial mismatch at the air-lens interface and unabsorbed backscattered waves. In case (iii), the incorporation of a PANI-based absorber behind the lens significantly enhances impedance matching. The absorber suppresses the back-reflected waves by converting them into heat through dielectric and magnetic losses, thus reducing standing wave formation and improving energy coupling into the antenna structure. This combined effect of the lens shaping the wavefront and the absorber mitigating reflections leads to the most favorable |S_11_| profile across the desired frequency band, confirming the effectiveness of the integrated lens-absorber system in achieving broadband impedance matching.

**Fig. 10:**
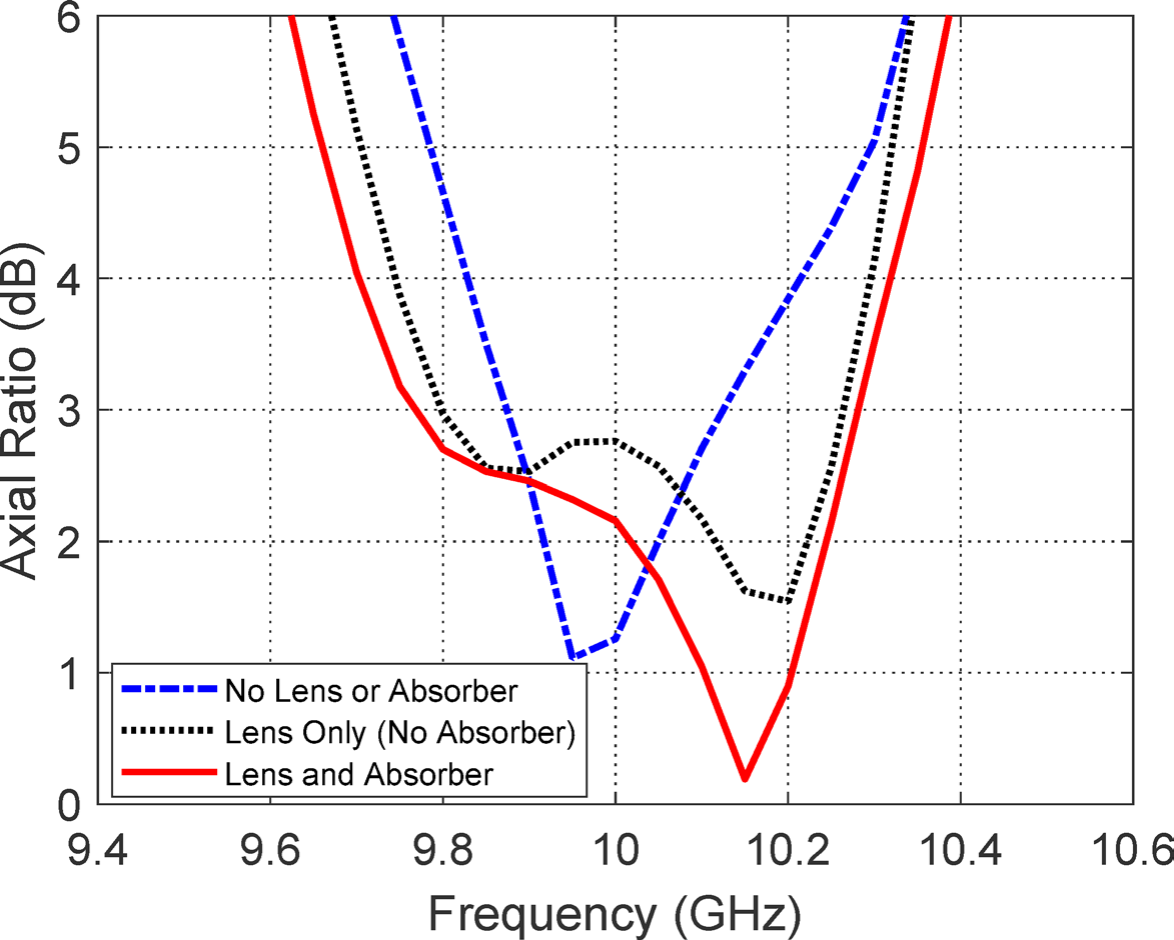
Comparison of the axial ratio frequency responses for three antenna configurations: (i) without lens or absorber, (ii) with lens only (no absorber), and (iii) with both lens and absorber. Among the three, the configuration combining both the lens and absorber achieves the most favorable axial ratio performance, maintaining values below the 3 dB threshold over the widest frequency range, thereby ensuring superior circular polarization purity.

Figure 10 presents the frequency-dependent axial ratio (AR) for three different configurations of the proposed antenna: (i) without lens or absorber, (ii) with lens only (no absorber), and (iii) with both lens and absorber. The axial ratio is a key indicator of the quality of circular polarization, with values below 3 dB generally indicating acceptable performance. In case (i), the absence of any external modifications leads to poor axial ratio performance across most of the frequency band due to uncontrolled phase and amplitude imbalances in the radiated fields, resulting in elliptically polarized waves with low purity. Introducing the dielectric lens in case (ii) significantly improves the AR response, as the lens helps to collimate and phase-correct the wavefronts emerging from the antenna, thereby improving the uniformity and symmetry of the radiated fields. This results in more consistent circular polarization, especially near the lens’ design frequency. In case (iii), the addition of the absorbing layer further enhances the axial ratio performance by suppressing unwanted reflections and side lobes that can introduce polarization distortion. The absorber minimizes backward waves and multiple reflections within the lens structure, leading to cleaner radiation patterns and enhanced axial ratio characteristics. Overall, the progressive improvement from case (i) to case (iii) demonstrates the complementary benefits of both the lens and absorber in refining the polarization behavior of the antenna.

**Fig. 11:**
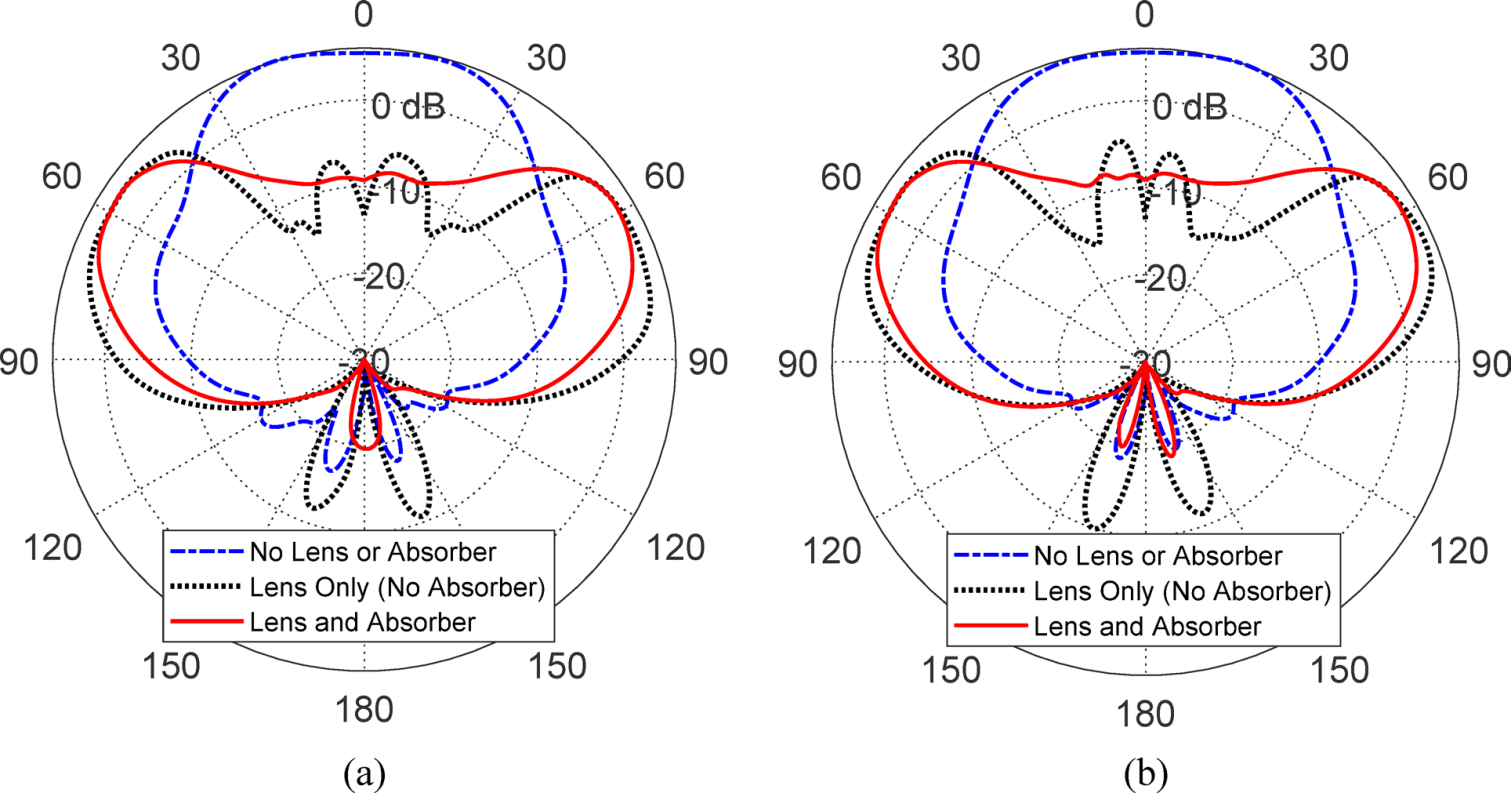
Comparison of the gain patterns in the elevation planes: (**a**) $\phi = 0^\circ$ and (**b**) $\phi = 90^\circ$, for three antenna configurations: (i) without lens or absorber, (ii) with lens only (no absorber), and (iii) with both lens and absorber. Among these configurations, the one incorporating both the lens and absorber yields the most desirable conical-shaped beam, characterized by enhanced beam symmetry and significantly reduced side lobes, making it well-suited for isoflux radiation applications.

Figure 11 presents the gain patterns in the elevation planes (a) $\phi = 0^\circ$ and (b) $\phi = 90^\circ$ for three antenna configurations: (i) without lens or absorber, (ii) with lens only (no absorber), and (iii) with both lens and absorber. In configuration (i), the antenna exhibits a conventional beam pattern with its maximum gain directed at nadir, indicating a non-conical radiation profile that is not suitable for isoflux applications. Configuration (ii), where only the lens is added, broadens the beam and shifts the gain away from nadir, producing a shape more consistent with isoflux requirements. However, this pattern still suffers from noticeable side lobes and gain ripples near the nadir direction, which degrade the uniformity and predictability of the radiation profile. In contrast, configuration (iii), which incorporates both the lens and the absorber, achieves a well-formed conical beam with a maximum gain of approximately 6 dBic at 63° off-nadir. This configuration eliminates undesired lobes and ripples, resulting in a smooth and symmetric radiation pattern that effectively satisfies the isoflux criteria for LEO satellite communication systems, where consistent coverage over a wide range of ground station elevation angles is essential.

### Parametric study for optimum dimensions

This section presents a systematic parametric study conducted to determine the optimum geometrical and excitation parameters that achieve the desired performance characteristics of the proposed isoflux antenna. The study is divided into two main parts. The first part (Sect. “Design of the polarizer and feeding ports”) focuses on minimizing input reflection ($\left| {S_{11} } \right|$) and achieving a low axial ratio over a wide frequency band, by optimizing the excitation probe length ($L_{P}$​), its position from the waveguide’s closed end ($L_{B}$​), and the distance of the external reflector disc from the waveguide aperture ($D_{A}$​). The second part (Sect. “Design of open-ended waveguide and reflecting disc”) aims at refining the antenna’s gain pattern to closely approximate the ideal isoflux shape suitable for LEO satellite coverage. This is done by varying key structural parameters such as the reflector disc diameter ($D_{B}$​), reflector position ($D_{A}$​), lens apex angle ($\theta_{A}$​), lens height ($H_{L}$), and lens base diameter ($D_{L}$​). Simulation results are used to identify the best values of each parameter, leading to a well-balanced design that satisfies both impedance and radiation requirements.

#### Parametric study for minimum reflection and axial ratio

**Fig. 12:**
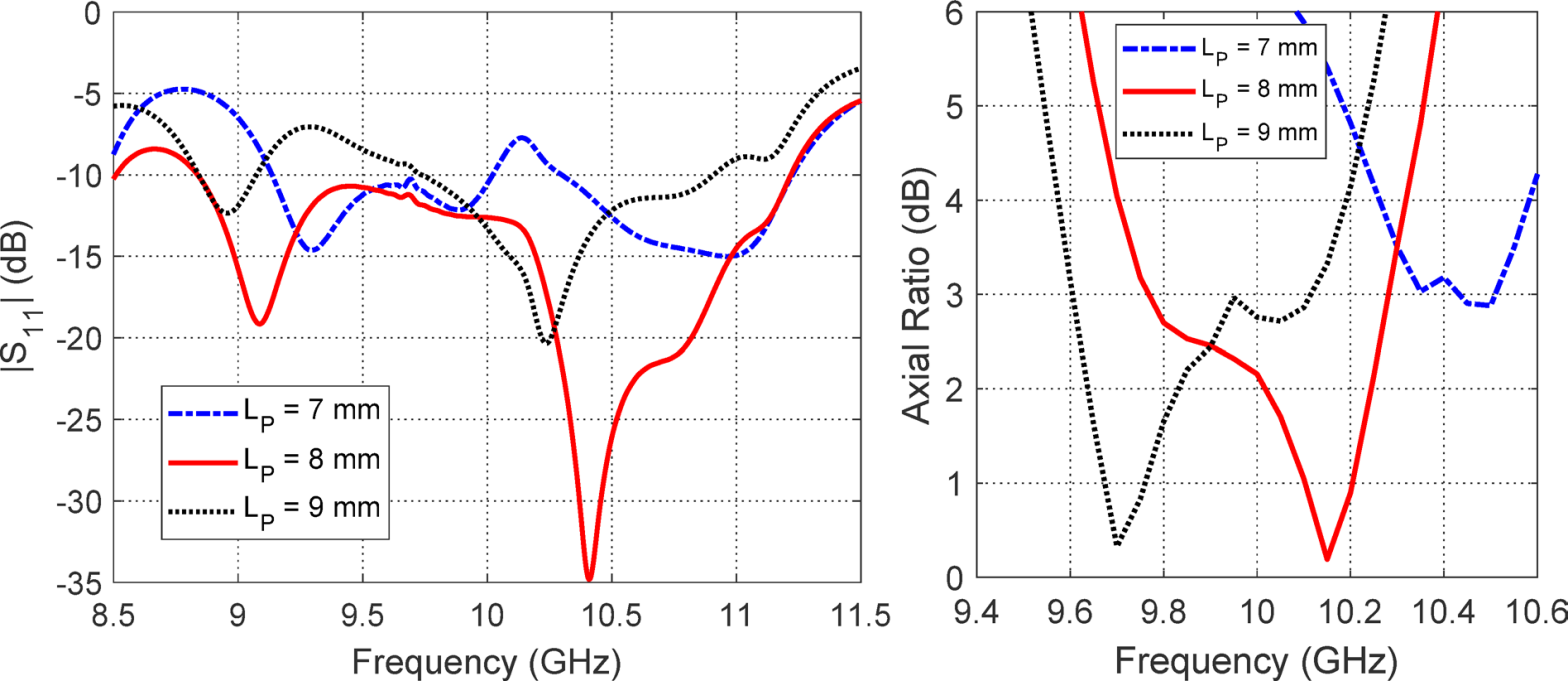
Variation of (**a**) $\left| {S_{11} } \right|$ and (**b**) axial ratio with frequency for different values of the excitation probe length ($L_{P}$).

**Fig. 13:**
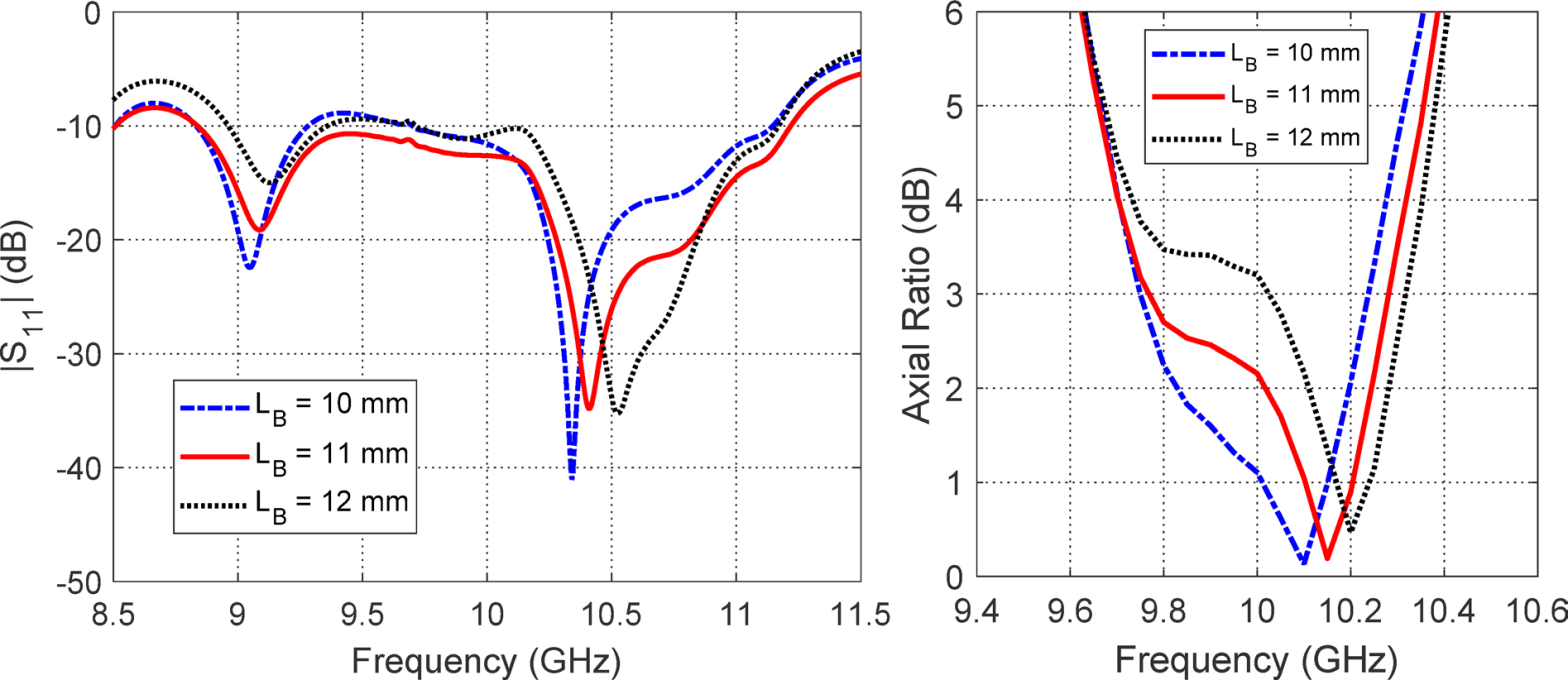
Variation of (**a**) $\left| {S_{11} } \right|$ and (**b**) axial ratio with frequency for different values of the excitation probe distance from the closed waveguide end ($L_{B}$).

**Fig. 14:**
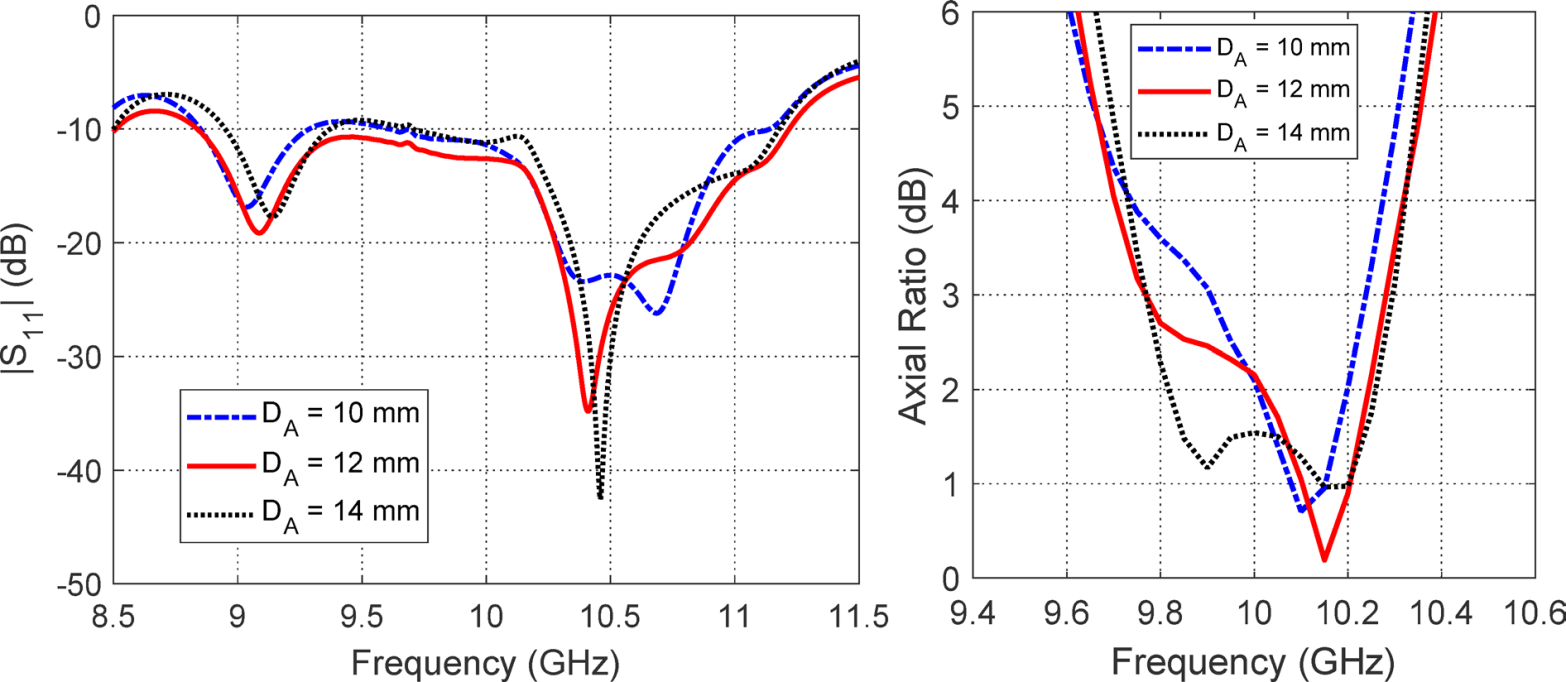
Variation of (**a**) $\left| {S_{11} } \right|$ and (**b**) axial ratio with frequency for different values of the distance of the reflecting circular disc from the open waveguide end ($D_{A}$).

Figures 12, 13 and 14 collectively present a parametric analysis of three key design variables that significantly influence the performance of the proposed isoflux antenna: the excitation probe length ($L_{P}$​), the probe’s axial position from the waveguide’s closed end ($L_{B}$​), and the axial placement of the external reflecting disc from the waveguide aperture ($D_{A}$​).

As shown in Fig. 12, varying the probe length directly impacts both impedance matching and polarization quality. Among the investigated values ($7{\text{ mm}}$, $8{\text{ mm}}$, and $9{\text{ mm}}$), the optimal performance is achieved at $L_{P} = 8{\text{ mm}}$, which yields the widest impedance-matching bandwidth $(\left| {S_{11} } \right| < - 10{\text{ dB}}$ over $8.75{-}11.25{\text{ GHz}}$) and the most favorable axial ratio below $3{\text{ dB}}$ across a broad frequency range.

Figure 13 explores the effect of the probe’s axial distance from the waveguide’s shorted end ($L_{B}$​), with tested values of $10{\text{ mm}}$, $11{\text{ mm}}$, and $12{\text{ mm}}$. The results indicate that $L_{B} = 11{\text{ mm}}$ provides the most balanced performance, offering improved impedance matching and polarization purity. This distance ensures proper excitation of orthogonal waveguide modes, which is essential for generating high-quality circular polarization and maintaining a good impedance match.

Figure 14 evaluates the influence of the reflector disc distance from the waveguide aperture ($D_{A}$​), with values of $10{\text{ mm}}$, $12{\text{ mm}}$, and $14{\text{ mm}}$ considered. The simulation shows that $D_{A} = 12\;{\text{mm}}$ results in optimal radiation characteristics by enhancing forward radiation, suppressing spurious modes at the aperture, and preserving axial ratio bandwidth.

Together, these parametric studies demonstrate the importance of careful tuning of $L_{P}$​, $L_{B}$​​, and $D_{A}$​ to achieve optimal antenna performance. The selected values, $D_{A} = 8\;{\text{mm}}$, $L_{B} = 11\;{\text{mm}}$, and $D_{A} = 12 {\text{mm}}$, are adopted in the final antenna design to ensure broadband impedance matching, high polarization purity, and well-shaped radiation patterns suitable for LEO satellite applications.

#### Parametric study for optimum shape of the gain pattern

**Fig. 15:**
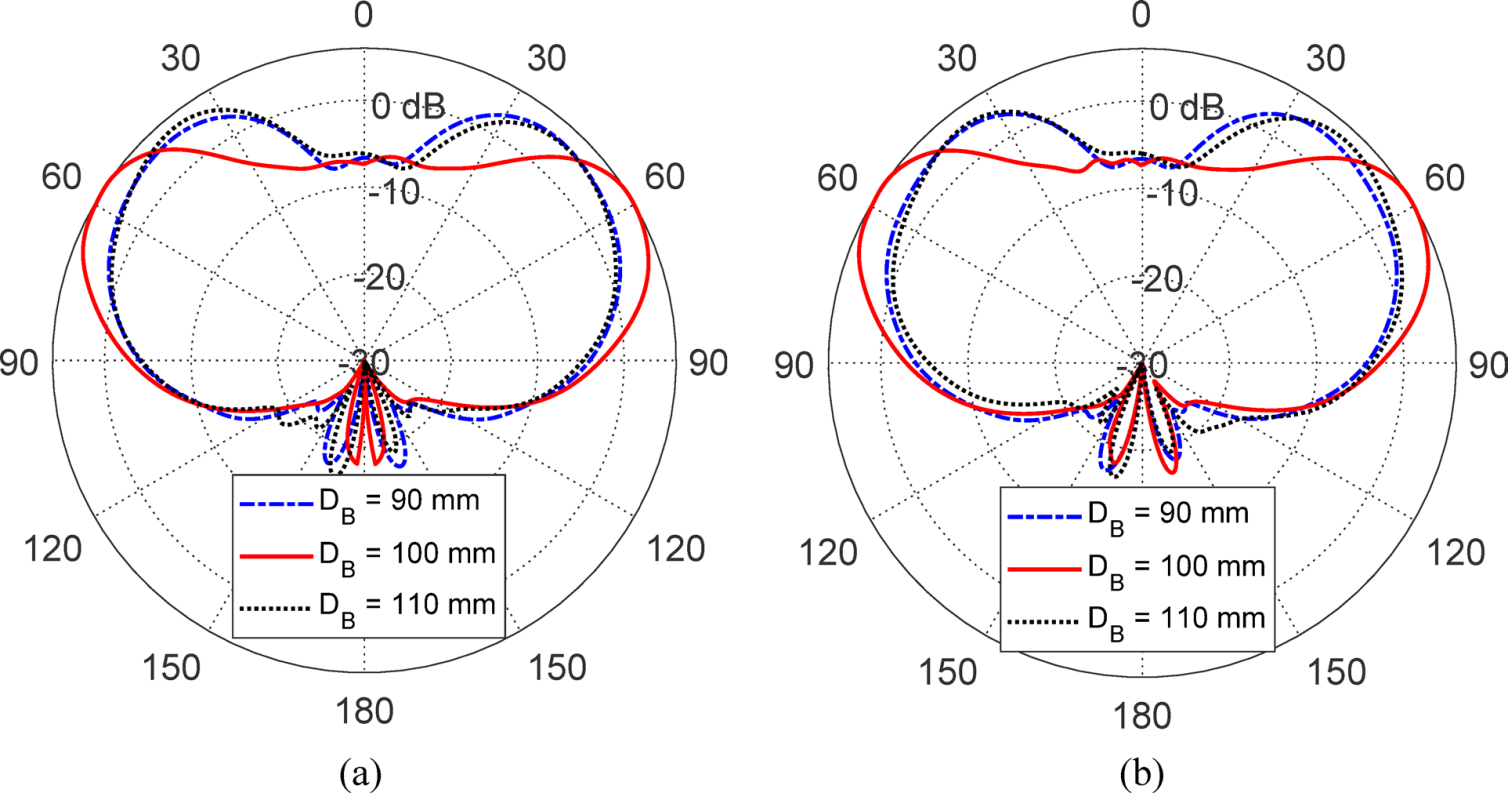
Effect of the reflector disc diameter ($D_{B}$) on the antenna gain pattern. Simulated results for $D_{B} = 90, \;100$, and $110 \; {\text{mm}}$. Elevation planes: (**a**) $\phi = 0^\circ$ and (**b**) $\phi = 90^\circ$.

**Fig. 16:**
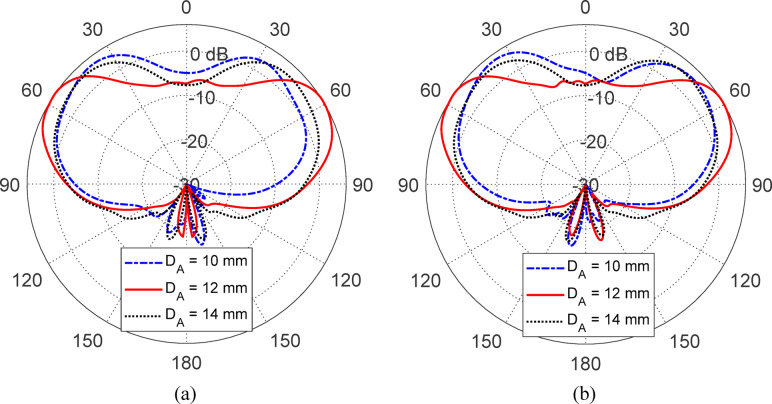
Variation of the antenna gain pattern with different reflector disc positions $(D_{A} = 10,{ }12,\;{\text{and}}\;14\;{\text{mm}}$) from the waveguide aperture. Elevation planes: (**a**) $\phi = 0^\circ$ and (**b**) $\phi = 90^\circ$.

**Fig. 17:**
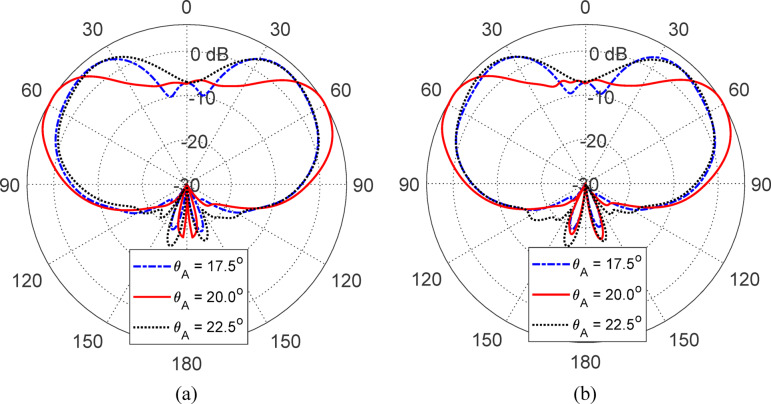
Influence of the lens half-apex angle ($\theta_{A} = 17.5^\circ ,20^\circ ,\;{\text{and}}\;22.5\;^\circ {\text{C}}$) on the antenna gain pattern. Elevation planes: (**a**) $\phi = 0^\circ$ and (**b**) $\phi = 90^\circ$.

**Fig. 18:**
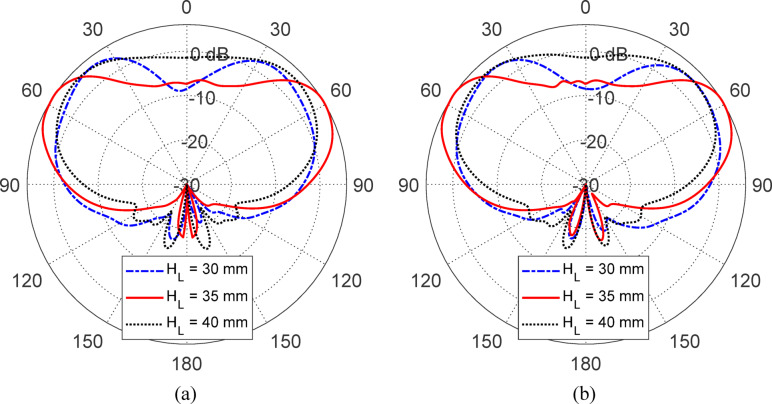
Effect of lens height ($H_{L} = 30,{ }35,{\text{ and }}40{\text{ mm}}$) on the simulated gain pattern of the antenna. Elevation planes: (**a**) $\phi = 0^\circ$ and (**b**) $\phi = 90^\circ$.

**Fig. 19:**
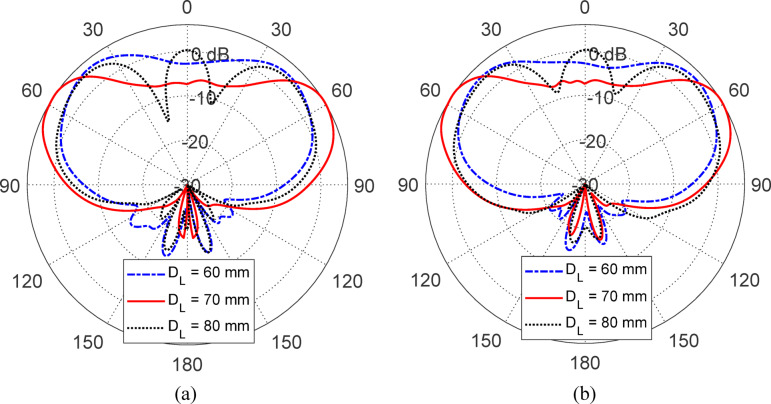
Variation of the antenna gain pattern with lens base diameter ($D_{L} = 60,{ }70,{\text{ and }}80{\text{ mm}}$). Elevation planes: (**a**) $\phi = 0^\circ$ and (**b**) $\phi = 90^\circ$.

Figures 15, 16, 17, 18 and 19 present a comprehensive parametric study aimed at optimizing the antenna’s gain pattern to closely match the desired isoflux-like distribution required for LEO satellite applications. The study evaluates the impact of five geometrical parameters: the reflector disc diameter ($D_{B}$​) (Fig. 15), the reflector position from the waveguide aperture ($D_{A}$​) (Fig. 16) , the lens half-apex angle ($\theta_{A}$​) (Fig. 17), the lens height ($H_{L}$​) (Fig. 18), and the lens base diameter ($D_{L}$​) (Fig. 19). For each parameter, three candidate values were simulated, and the results consistently show that the middle value in each set, $D_{B} = 100 {\text{mm}}$, $D_{A} = 12{\text{ mm}}$, $\theta_{A} = 20^\circ$, $H_{L} = 35\;{\text{mm}}$, and $D_{L} = 70{\text{ mm}}$, produces a gain pattern that most accurately conforms to the desired broad conical coverage centered at ± 63° from nadir. These values are thus adopted in the final design to ensure effective spatial coverage and beam uniformity across the satellite’s footprint.

## Preparation of PANI nano-material

In high-frequency satellite communication systems, especially at X-band, maintaining high polarization purity and wide bandwidth with compact and lightweight antenna structures is a critical challenge. To address this, a PANI-based nano-material was employed as a microwave absorber in the proposed waveguide lens antenna. Positioned between the dielectric lens and the metallic backing plate at the open end of the waveguide, this absorbing layer plays a dual role: minimizing surface wave reflections and suppressing unwanted resonances, thereby significantly improving the axial ratio, gain, and polarization purity of the antenna across its operational band.

### Synthesis of polyaniline (PANI)

A standard oxidative polymerization method was used to synthesize polyaniline (emeraldine salt form). Aniline hydrochloride (purum; $2.59{\text{ g}}$, $20{\text{ mmol}}$) was dissolved in distilled water to prepare $50{\text{ mL}}$ of $0.2{\text{ M solution}}$. Separately, ammonium peroxydisulfate (purum; $5.71{\text{ g}}$, $25{\text{ mmol}}$) was dissolved in $50{\text{ mL}}$ of distilled water to obtain a $0.25{\text{ M}}$ solution. After allowing both solutions to stand at room temperature ($\sim 18{-}24{ }^\circ {\text{C}}$) for 1 h, they were mixed under brief stirring in a beaker and left undisturbed to polymerize overnight.The resulting green PANI precipitate was filtered and washed successively with three $100$-${\text{mL}}$ portions of $0.2{\text{ M HCl}}$ and acetone. The filtered product was air-dried and then further dried under vacuum at $60{ }^\circ {\text{C}}$. These samples are referred to as “standard” PANI. In additional runs, polymerization was conducted in an ice bath ($0{-}2{ }^\circ {\text{C}}$), and the acidity was increased in some samples by replacing $10{\text{ mL}}$ of water with $10{\text{ mL}}$ of $10{\text{ M HCl}}$. For doped samples, $0.05{\text{ g}}$ of PANI was mixed with $0.15{\text{ g}}$ of DBSA in a beaker, then dissolved in chloroform to obtain 50 mL of homogeneous solution.

### X-ray diffraction (XRD) analysis

**Fig. 20:**
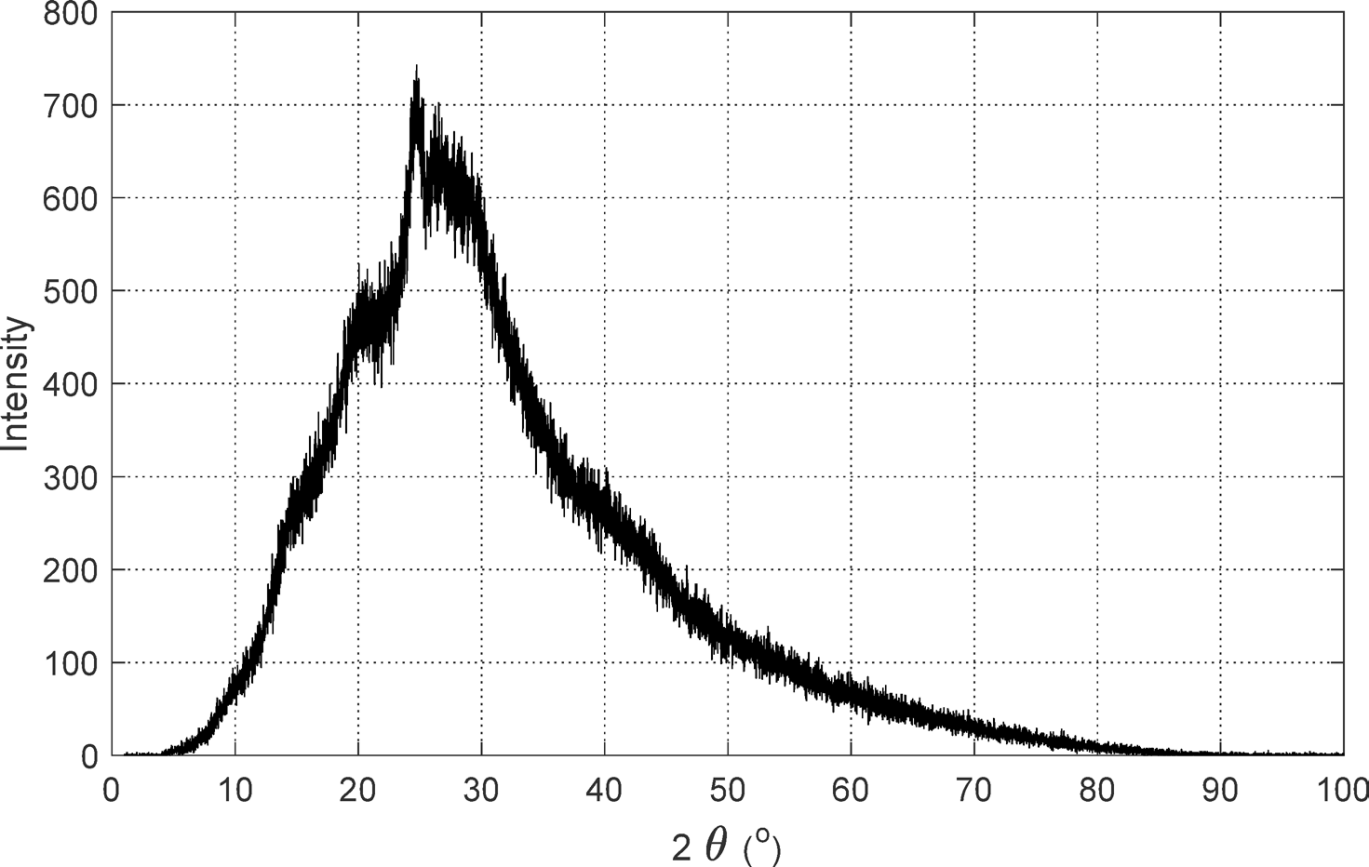
Powder XRD pattern of synthesized PANI using ${\text{Cu K}}\upalpha$ radiation ($\lambda = 1.54060$ Å).

The crystalline structure of synthesized PANI was analyzed using powder XRD on a Philips diffractometer with ${\text{Cu K}}\upalpha$ radiation ($\lambda = 1.54060$ Å); as representd in Fig. 20. The scanning was performed from $10^\circ$ to $90^\circ$ in $2\theta$ at a rate of $10^\circ /{\text{min}}$. A broad peak observed at $2\theta \approx 25.53^\circ$ indicated the amorphous nature of the PANI samples, consistent with literature reports^17–22^.

### Scanning electron microscopy (SEM)

**Fig. 21:**
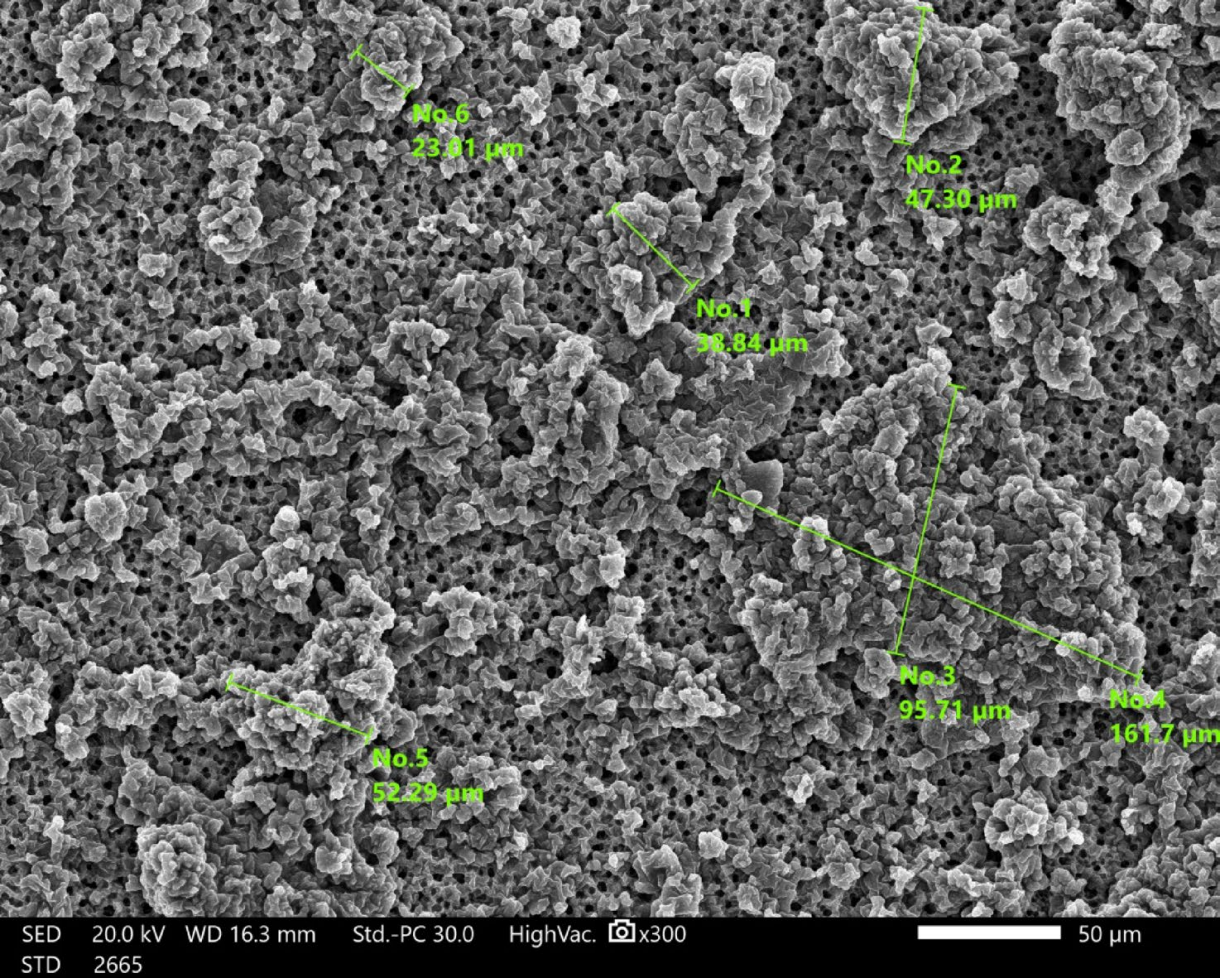
SEM image of DBSA-doped PANI at 300 × magnification. The image shows a relatively uniform spherical granular morphology with particle diameters ranging from $100{-}200{\text{ nm}}$.

SEM image of DBSA-doped PANI is presented in Fig. 21. This figure shows the PANI sample exhibiting a relatively uniform spherical granular morphology, with average particle diameters ranging from 100 to 200 nm. This globular structure is attributed to coiled polymer chains templated by DBSA micelles.

**Fig. 22:**
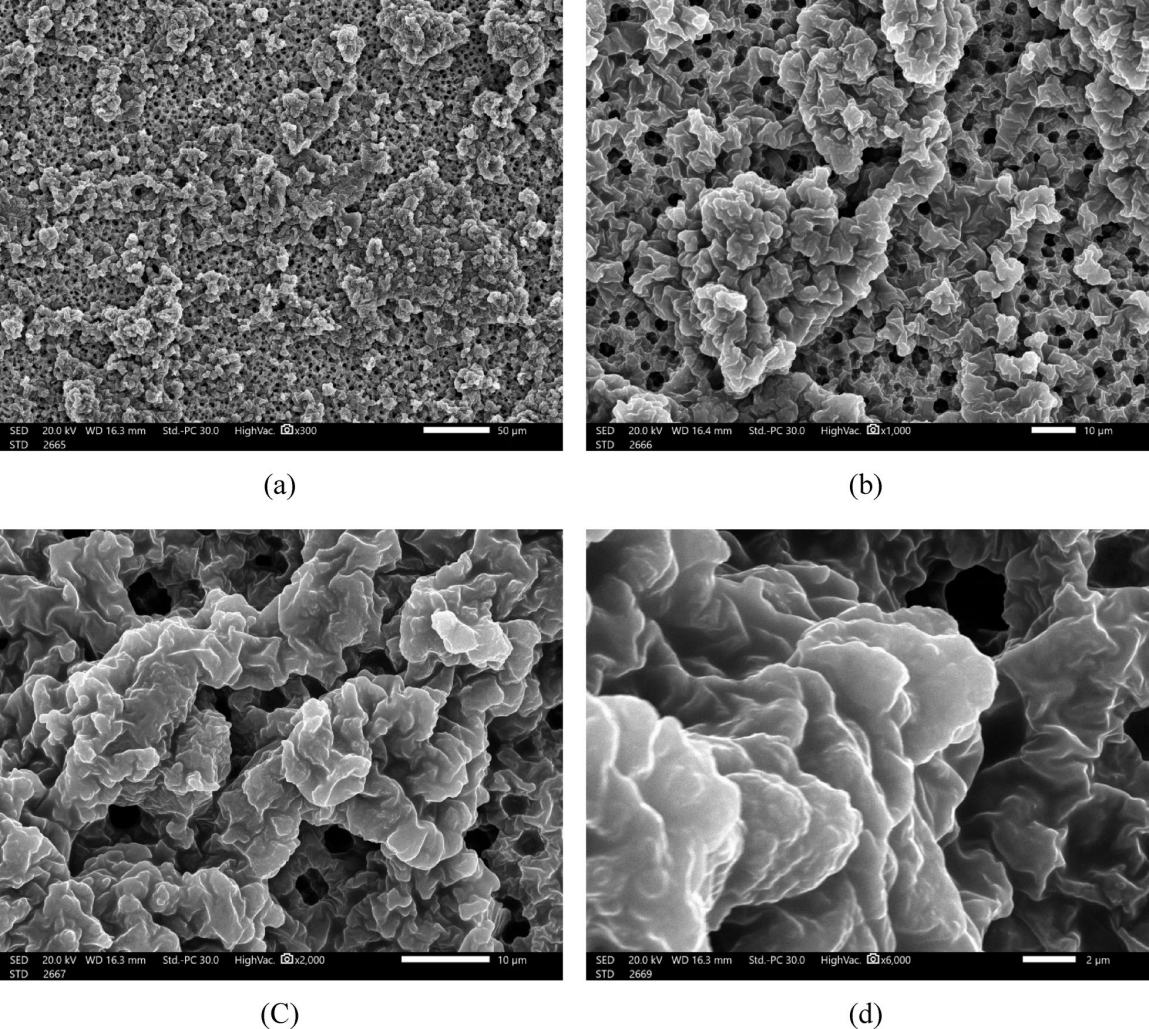
SEM images of DBSA-doped PANI captured at increasing magnifications: (**a**) 300x, (**b**) 1000x, (**c**) 2000x, and (**d**) 6000x. The micrographs reveal a characteristic globular morphology, which is attributed to the self-assembly of coiled polyaniline chains templated by DBSA micelles.

The SEM analysis in Fig. 22 highlights the unique surface morphology of DBSA-doped PANI, revealing a globular and porous microstructure across multiple magnification levels. This morphology arises from the micelle-templated polymerization process, where DBSA acts both as a dopant and a surfactant. The presence of microscale porosity and coiled domains contributes significantly to the material’s effective permittivity and microwave absorption characteristics. Such structural features are advantageous for suppressing surface waves and back-lobe radiation when the material is used as a lossy absorber in the antenna structure, ultimately improving gain uniformity and polarization purity. The porous and semi-crystalline texture observed at higher magnifications is indicative of a heterogeneous structure favorable for electromagnetic wave absorption. Imaging was performed under a secondary electron detector (SED) at an accelerating voltage of 20.0 kV and a working distance (WD) of 16.3 mm in high vacuum mode.

## Antenna fabrication and measurement

This section presents the practical realization and experimental validation of the proposed isoflux antenna design. In Sect. “Antenna fabrication”, the fabrication process is described in detail, highlighting the sequential assembly of the antenna components, including the dual-port polarizer, open-ended waveguide, reflector disc, dielectric lens, and the PANI nano-material absorber. Precision machining and accurate alignment of each part are crucial to preserving the design specifications and ensuring optimal electromagnetic performance. Section “Measurement of the reflection coefficient and ports isolation” focuses on the measurement setup used to evaluate key antenna parameters, particularly the input reflection coefficient ($\left| {S_{11} } \right|$ and $\left| {S_{22} } \right|$) and isolation between the two excitation ports ($\left| {S_{12} } \right|$ and $\left| {S_{21} } \right|$). Measurements are conducted across the X-band frequency range using a calibrated vector network analyzer in a controlled laboratory environment, and the results are compared with simulation data to verify the performance of the fabricated antenna.

### Antenna fabrication

**Fig. 23:**
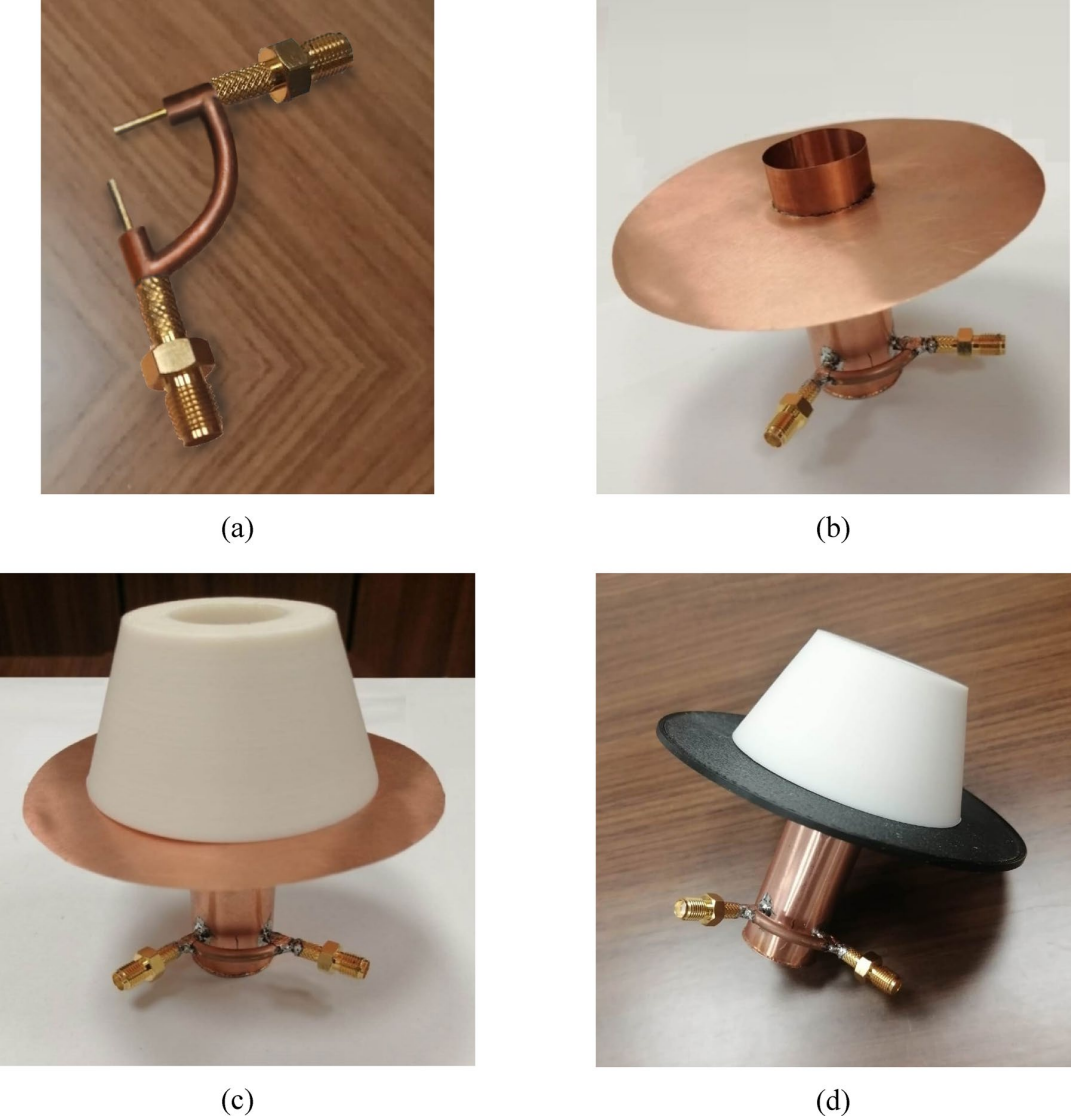
Antenna fabrication process: (**a**) Fabricated dual-port polarizer. (**b**) Assembly of the dual-port polarizer with the open-ended circular waveguide and attachment of the metallic reflector disc. (**c**) Placement of the dielectric lens on top of the reflector disc without the absorber. (**d**) Final antenna configuration after inserting the PANI absorber disc between the lens base and the reflector disc.

The fabrication process of the proposed isoflux antenna is carried out in sequential steps, as illustrated in Fig. 23. First, the dual-port polarizer is fabricated using precision machining techniques. The polarizer includes two orthogonally oriented probe ports designed to excite orthogonal modes within the circular waveguide. The probes are carefully dimensioned and placed at optimized locations based on the parametric design study to ensure circular polarization and impedance matching as represented in Fig. 20a.

In the next step, the dual-port polarizer is assembled with the circular waveguide, which serves as the primary radiating aperture. The circular waveguide is fabricated from a conductive metallic material, and the polarizer is securely mounted to its closed end. A circular reflector disc is then welded to the outer wall of the waveguide at a specific distance from its open end, as determined in the design stage, to control the radiation characteristics and suppress unwanted back-lobe radiation as displayed in Fig. 23b.

Following this, the dielectric lens is positioned concentrically on top of the reflector disc, aligning it with the waveguide’s open aperture. The lens is precision-molded from Teflon, a low-loss dielectric material with a relative permittivity of 2. It is shaped into a top-cut conical profile with optimized dimensions to generate the desired broad conical beam. In this intermediate stage, the absorber is not yet included as shown in Fig. 23c.

Finally, the PANI nano-material absorbing disc is inserted between the lens base and the reflector disc, completing the final antenna structure as displayed in Fig. 23d. The absorber disc is fabricated by coating a thin circular plate with synthesized PANI, which exhibits favorable microwave absorption characteristics due to its lossy behavior and micro-porous morphology. The inclusion of this layer enhances circular polarization purity, suppresses surface currents, and improves gain performance by minimizing backward radiation and reflections at the lens–waveguide interface.

This step-by-step assembly process ensures structural integrity and electromagnetic optimization, translating the design into a fully functional prototype ready for measurement and evaluation.

### Measurement of the reflection coefficient and ports isolation

**Fig. 24:**
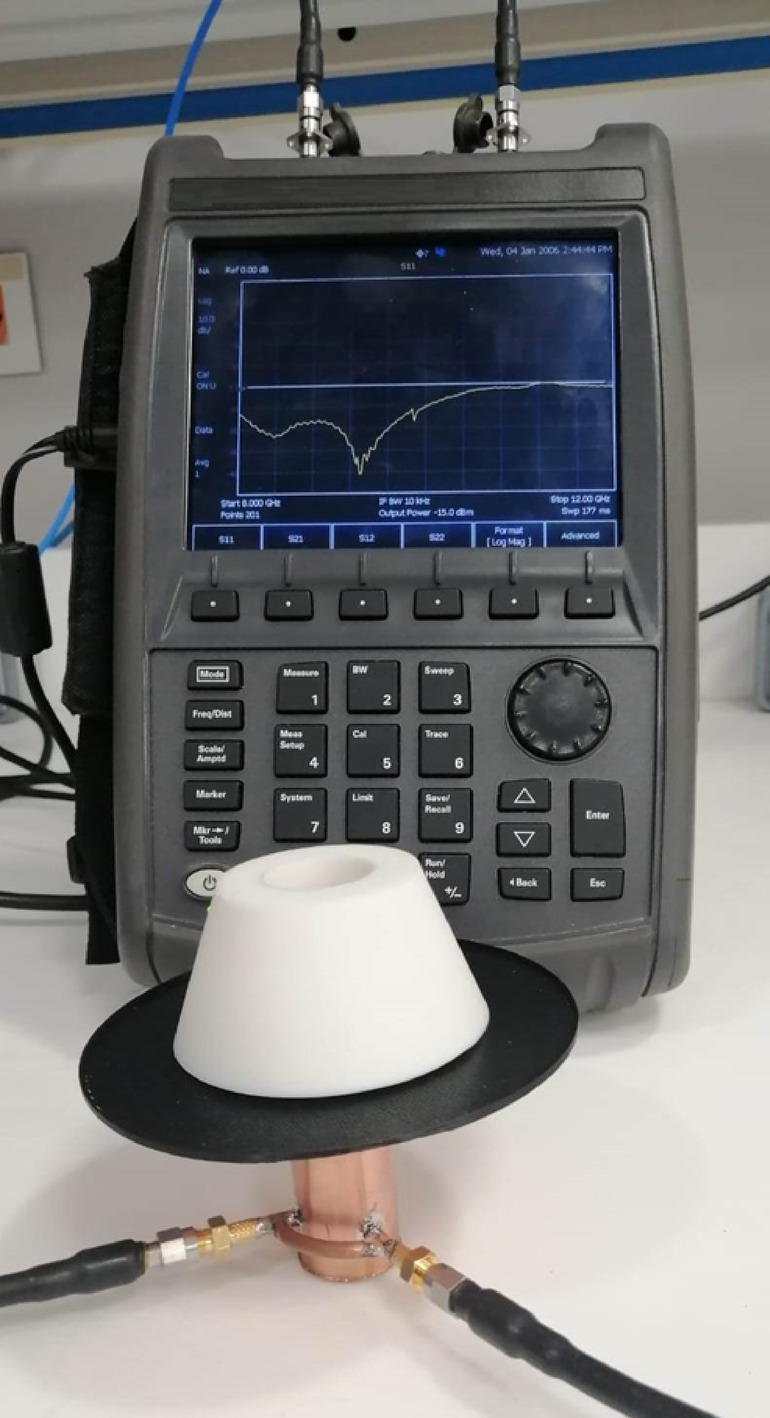
Measurement setup for evaluating the reflection coefficient ($S_{11}$) and port-to-port isolation ($\left| {S_{21} } \right|$) of the antenna using the Agilent FieldFox VNA, model N9918A.

Figure 24 illustrates the measurement setup employed to evaluate the reflection coefficient ($S_{11}$) and the isolation between the antenna ports $(\left| {S_{21} } \right|$) using the Agilent FieldFox Vector Network Analyzer (VNA), model N9918A. This setup enables accurate characterization of the antenna’s input impedance and mutual coupling behavior across the frequency band of interest. The reflection coefficient ($S_{11}$) measurement is crucial for assessing impedance matching at each port, which directly influences the efficiency of power transfer and the overall performance of the antenna. Meanwhile, the isolation ($\left| {S_{21} } \right|$) quantifies the degree of electromagnetic coupling between the two ports, with lower values indicating better port isolation and minimal interference in a dual-polarized or MIMO configuration. The use of a portable yet precise instrument like the FieldFox VNA ensures reliable field and laboratory measurements, contributing to the comprehensive validation of the antenna’s design parameters.

### Measurement of the far field

**Fig. 25:**
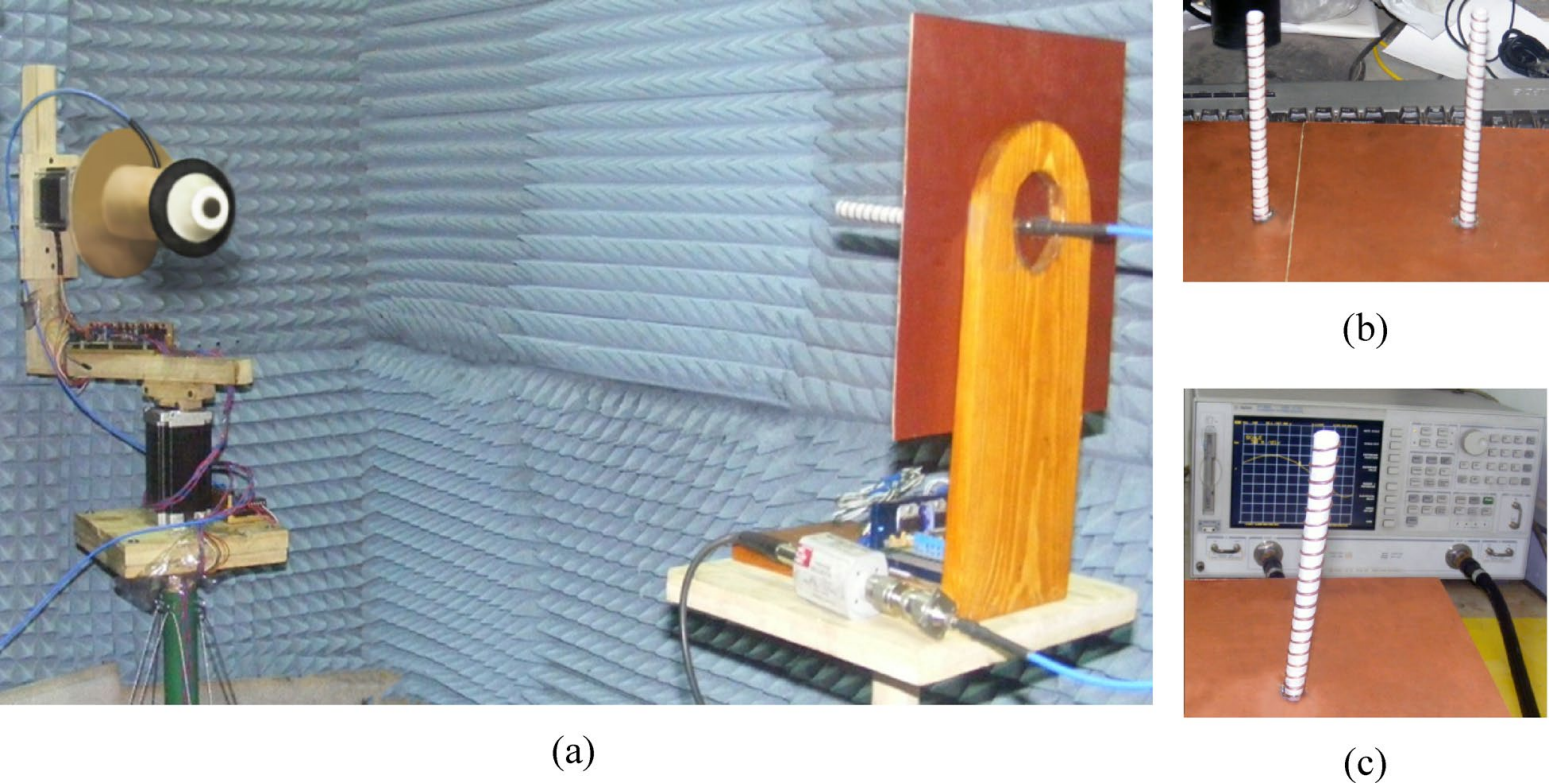
Experimental setup and components used for far-field characterization of the proposed isoflux antenna: (**a**) measurement setup inside the shielded anechoic chamber using the Agilent FieldFox VNA (N9918A); (**b**) fabricated LHCP and RHCP reference helical antennas; (**c**) VNA-based validation of the reference antennas for use in polarization and axial ratio measurements.

Figure 25 provides a comprehensive overview of the experimental setup used for characterizing the far-field performance of the proposed isoflux antenna. As shown in Fig. 25a, the measurements were conducted in a shielded anechoic chamber to eliminate external interference and suppress unwanted reflections, thereby ensuring accurate evaluation of the antenna’s radiation properties. The setup includes an Agilent FieldFox vector network analyzer (VNA), model N9918A, which is used to measure S-parameters and extract far-field characteristics such as the radiation pattern, gain, axial ratio, and cross-polarization levels.

To evaluate the circular polarization quality of the isoflux antenna, two broadband reference helical antennas with well-defined polarization purity are employed—one configured for LHCP and the other for RHCP. These reference antennas, which are custom-fabricated for this purpose as shown in Fig. 25b, possess excellent axial ratio performance around 10 GHz and serve as polarization filters during the measurement process. By orienting the appropriate helical antenna in the receiving path, the co-polarized (LHCP or RHCP) and cross-polarized components of the radiated field from the device under test can be selectively captured. This enables accurate determination of the axial ratio, a key metric for assessing circular polarization quality.

Figure 25c illustrates the preliminary characterization of the reference antennas using the VNA. This step is essential to establish a reliable performance baseline for the LHCP and RHCP reference antennas, ensuring that the polarization measurements of the isoflux antenna are conducted with high fidelity. The collected reference data is also used for calibration and normalization purposes during post-processing of the measured results. Overall, Fig. 25 highlights the thorough experimental methodology adopted to validate the polarization and radiation characteristics of the proposed antenna design.

## Results and discussions

This section presents and analyzes the key findings of this study, encompassing both the material characterization and the antenna performance evaluation. Section “Electromagnetic properties of PANI powder” focuses on the electromagnetic properties of DBSA-doped PANI powder, which is explored as a microwave absorbing material for improving the radiation characteristics of the proposed antenna. Through detailed measurements of frequency-dependent permittivity and permeability, the intrinsic dielectric and magnetic loss behavior of the material is established, providing insights into its absorption capabilities. Section “Isoflux antenna characteristics” then examines the radiation performance of the proposed dual-port isoflux circularly polarized antenna, integrating the PANI-based absorber to enhance pattern shaping and reduce back-reflections. This section presents both simulated and measured data for key antenna parameters, including impedance matching, port isolation, axial ratio, and gain patterns, demonstrating the effectiveness of the design for LEO satellite applications.

### Electromagnetic properties of PANI powder

**Fig. 26:**
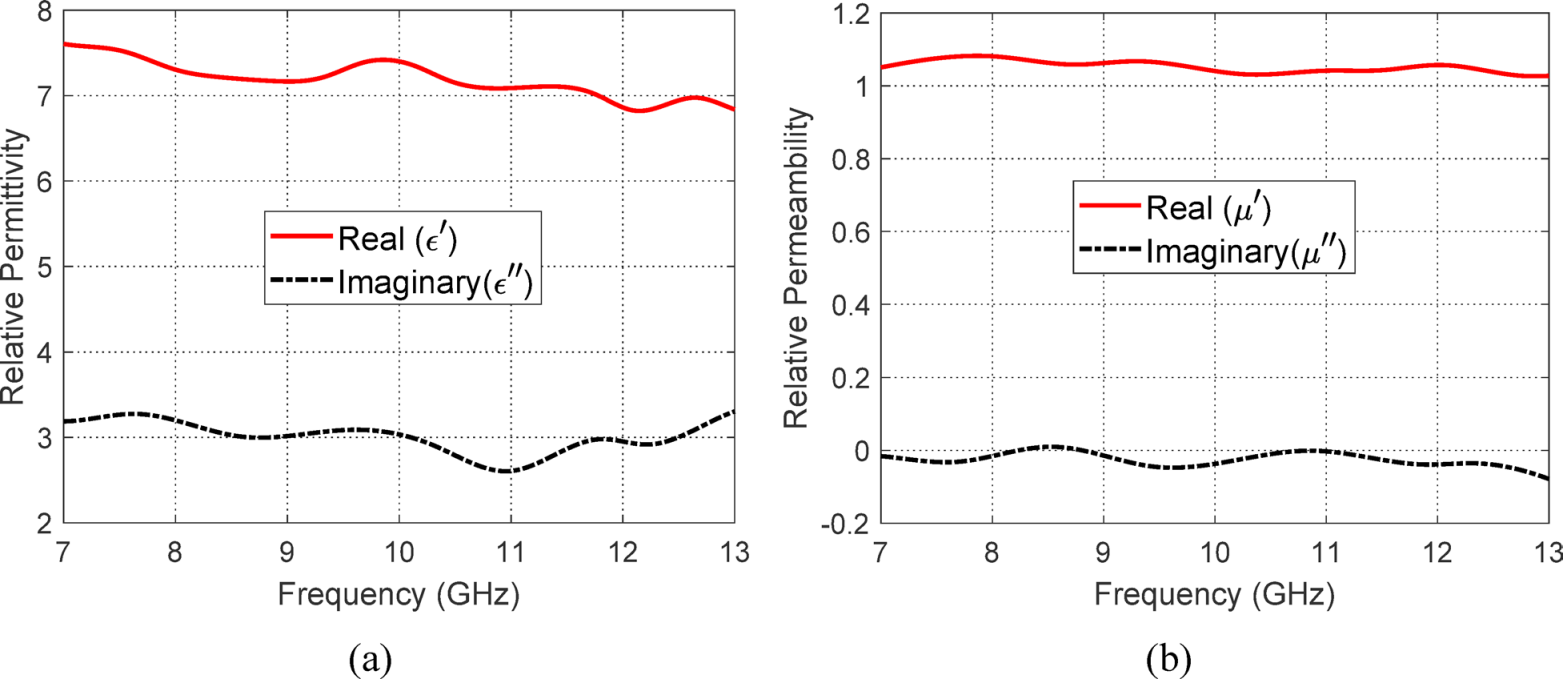
Frequency-dependent complex relative permittivity and permeability of DBSA-doped PANI powder. (**a**) Real and imaginary parts of the relative permittivity ($\varepsilon ^{\prime}$ and $\varepsilon$″), and (**b**) real and imaginary parts of the relative permeability ($\mu ^{\prime}$ and $\mu ^{\prime \prime}$).

**Fig. 27:**
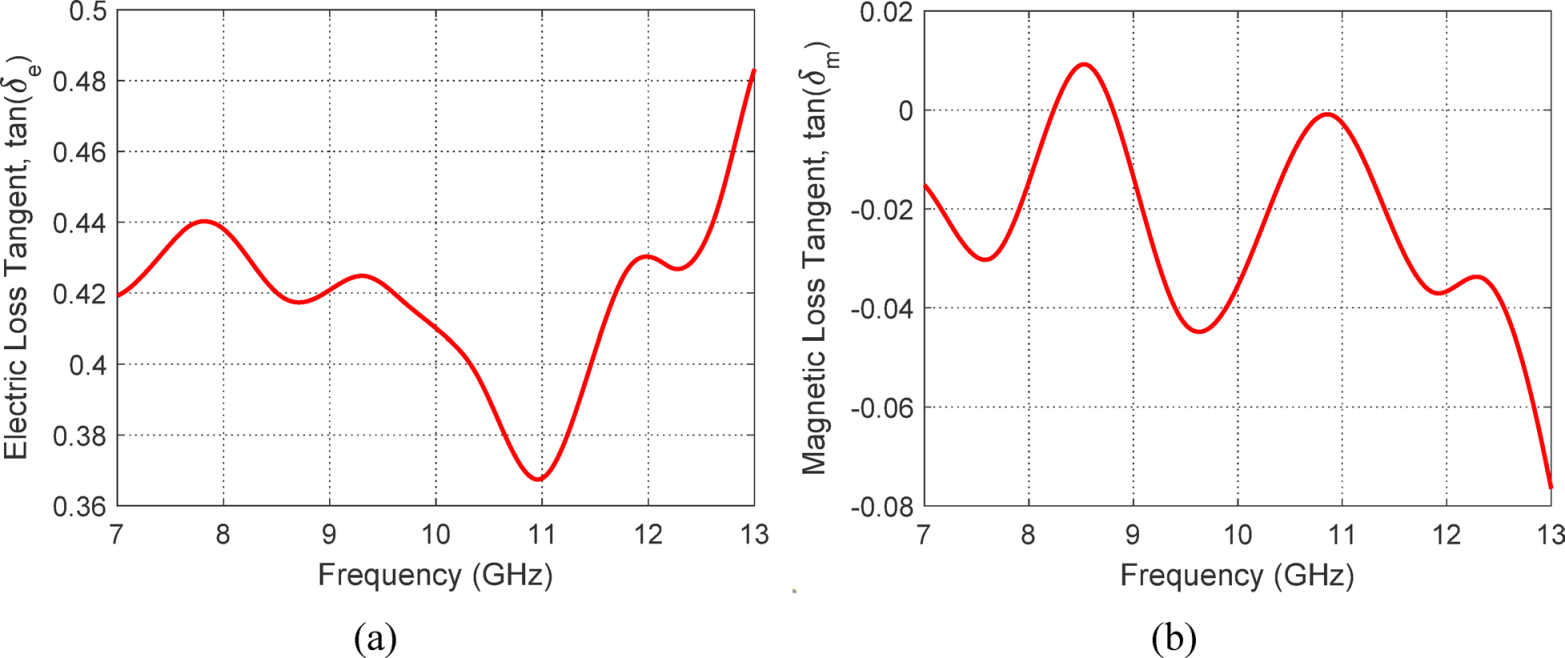
Dielectric and magnetic loss factors of DBSA-doped PANI powder over the 7–13 GHz frequency range: (**a**) dielectric loss tangent, $\tan \left( {\updelta _{\text{e}} } \right) = \upvarepsilon ^{\prime \prime} /\upvarepsilon^{\prime}$, and (**b**) magnetic loss tangent, $\tan \left( {\updelta _{\text{m}} } \right) = \upmu ^{\prime\prime}/\upmu^{\prime}.$ This strong dielectric–magnetic loss correlation highlights the capability of doped PANI structures to respond to both electric and magnetic field variations, which is critical for efficient microwave absorption.

This section presents a comprehensive characterization of the electromagnetic properties of DBSA-doped PANI powder, which is investigated as a candidate material for use in high-performance microwave absorber sheets integrated into the proposed antenna system. The motivation for selecting doped PANI lies in its intrinsic conductive polymer structure, which can be engineered to exhibit both significant dielectric and magnetic responses at microwave frequencies. Figures 25 and 26 detail the complex relative permittivity and permeability of the material, along with the corresponding dielectric and magnetic loss tangents, over the 7–13 GHz frequency range. These results demonstrate a strong correlation between electric and magnetic loss mechanisms, indicative of the material’s capacity to interact effectively with both components of incident electromagnetic waves. Such dual-loss behavior is highly desirable for broadband microwave absorption. Figure 27 further evaluates the absorptive performance of a PANI sheet, comparing free-standing and metal-backed configurations. The enhanced absorptivity observed in the metal-backed case confirms the potential of DBSA-doped PANI as an efficient lossy coating for antenna systems, particularly in applications where suppression of unwanted reflections and improved radiation control are essential—such as in the proposed isoflux antenna for LEO satellite communication.

#### Complex permeability and permittivity

Figure 26 illustrates the frequency-dependent behavior of the complex relative permittivity ($\varepsilon ^{\prime}$ and $\varepsilon ^{\prime \prime}$) and permeability ($\mu ^{\prime}$ and $\mu ^{\prime \prime}$) of DBSA-doped PANI powder measured over the 7–13 GHz range. The real parts ($\varepsilon ^{\prime}$ and $\mu ^{\prime}$) represent the material’s ability to store electric and magnetic energy, respectively, while the imaginary parts ($\varepsilon ^{\prime \prime}$ and $\mu ^{\prime \prime}$) reflect the corresponding energy losses due to dielectric and magnetic dissipation. The dielectric and magnetic loss tangents, defined as ${\text{tan}}\left( {\delta_{{\text{e}}} } \right) = \varepsilon ^{\prime \prime} /\varepsilon ^{\prime}$ and ${\text{tan}}\left( {\delta_{{\text{m}}} } \right) = \mu ^{\prime \prime} /\mu$, are critical indicators of the material’s efficiency in attenuating incident microwave energy. As evident in Fig. 27a and b, both loss tangents maintain relatively high and stable values across the measured frequency range, suggesting effective and broad microwave attenuation performance. This behavior is attributed to the presence of polarization relaxation mechanisms within the doped PANI structure, which enable simultaneous interactions with electric and magnetic field components of electromagnetic waves. The alignment of dielectric and magnetic losses across the band implies that the DBSA-doped PANI exhibits a synergistic dual-loss mechanism, enhanced by localized polarization dynamics and conductive pathways, making it a strong candidate for efficient and broadband microwave absorbing applications.

To accurately model the PANI material properties in CST Studio Suite, we defined the material using its measured complex permittivity and permeability spectra. These frequency-dependent material parameters were incorporated into the CST simulation by using the dispersive material modeling feature of the time-domain solver. Specifically, we employed interpolated broadband material data, which allows CST to apply a causal fitting algorithm (such as a Lorentz or Debye model) internally, enabling accurate time-domain simulation of dispersive behavior across the frequency range of interest. This implementation ensures a faithful representation of the absorber’s electromagnetic response, and the material was assigned to the corresponding regions (e.g., surface backing or volume) in the simulation model accordingly.

#### Electromagnetic absorptivity

**Fig. 28:**
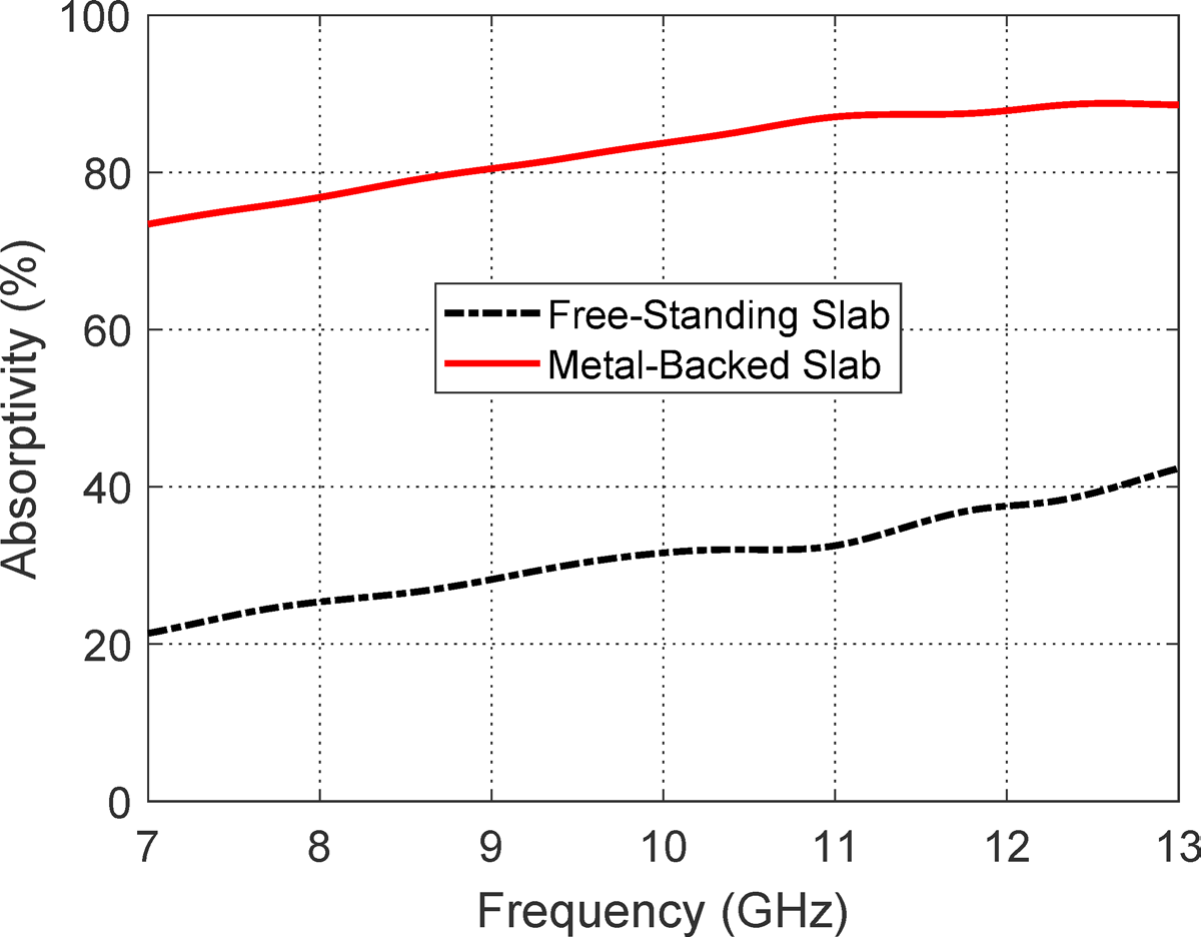
Absorptivity versus frequency for a PANI sheet of 1.5 mm thickness: comparison between free-standing configuration and metal-backed configuration. The metal-backed configuration exhibits higher absorptivity across the frequency range due to enhanced internal reflections and increased effective path length for electromagnetic dissipation.

When a PANI sheet is exposed to electromagnetic waves, its ability to absorb energy, termed absorptivity, depends significantly on whether it is free-standing or backed by a conducting surface. Figure 28 illustraes the dependence of the absorptivity on the frequency frequency for a PANI sheet of 1.5 mm thickness showing a comparison between free-standing configuration and metal-backed configuration. In a free-standing configuration, the incident wave partially reflects off the front surface, partially transmits through the material, and only a portion of the energy is absorbed as the wave passes through once. This limits the interaction length within the lossy material and allows some energy to escape, reducing overall absorption. However, when the same PANI sheet is backed by a perfect electric conductor (PEC), the behavior changes drastically. The PEC prevents any transmission, so the entire wave is reflected back into the PANI sheet after initially entering it. This effectively doubles the path length that the wave travels within the absorbing medium, allowing more energy to be dissipated through dielectric and magnetic losses. Additionally, the interference between the forward and reflected waves inside the slab enhances the local field intensities, which further increases the absorption. As a result, the metal-backed configuration not only eliminates transmission losses but also promotes stronger internal reflections and field interactions, leading to significantly higher absorptivity compared to the free-standing slab.

### Isoflux antenna characteristics

**Fig. 29:**
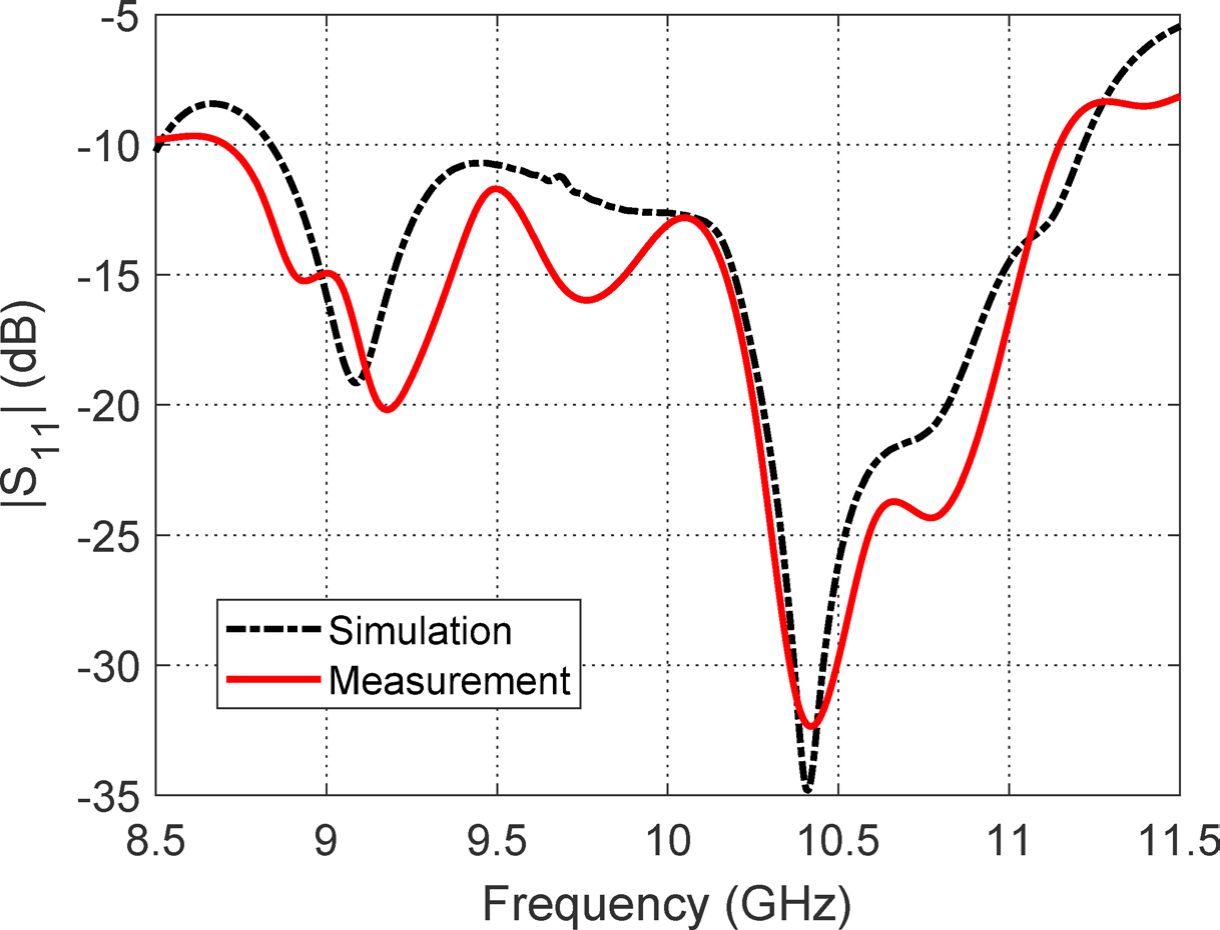
Measured and simulated frequency responses of $\left| {S_{11} } \right|$ at port 1 for RHCP.

**Fig. 30:**
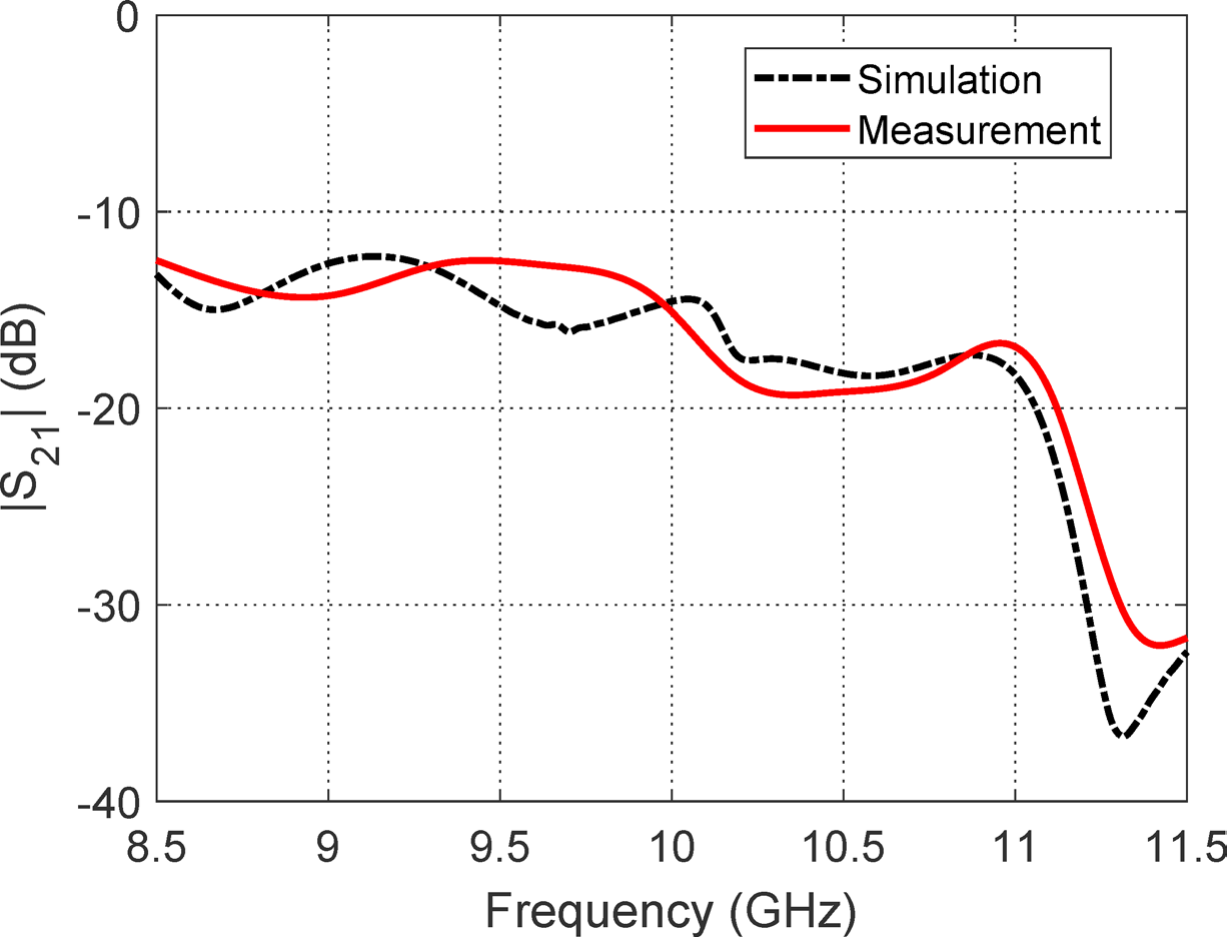
Measured and simulated frequency response of the transmission coefficient (|S₂₁|), illustrating the isolation performance between the two antenna ports.

**Fig. 31:**
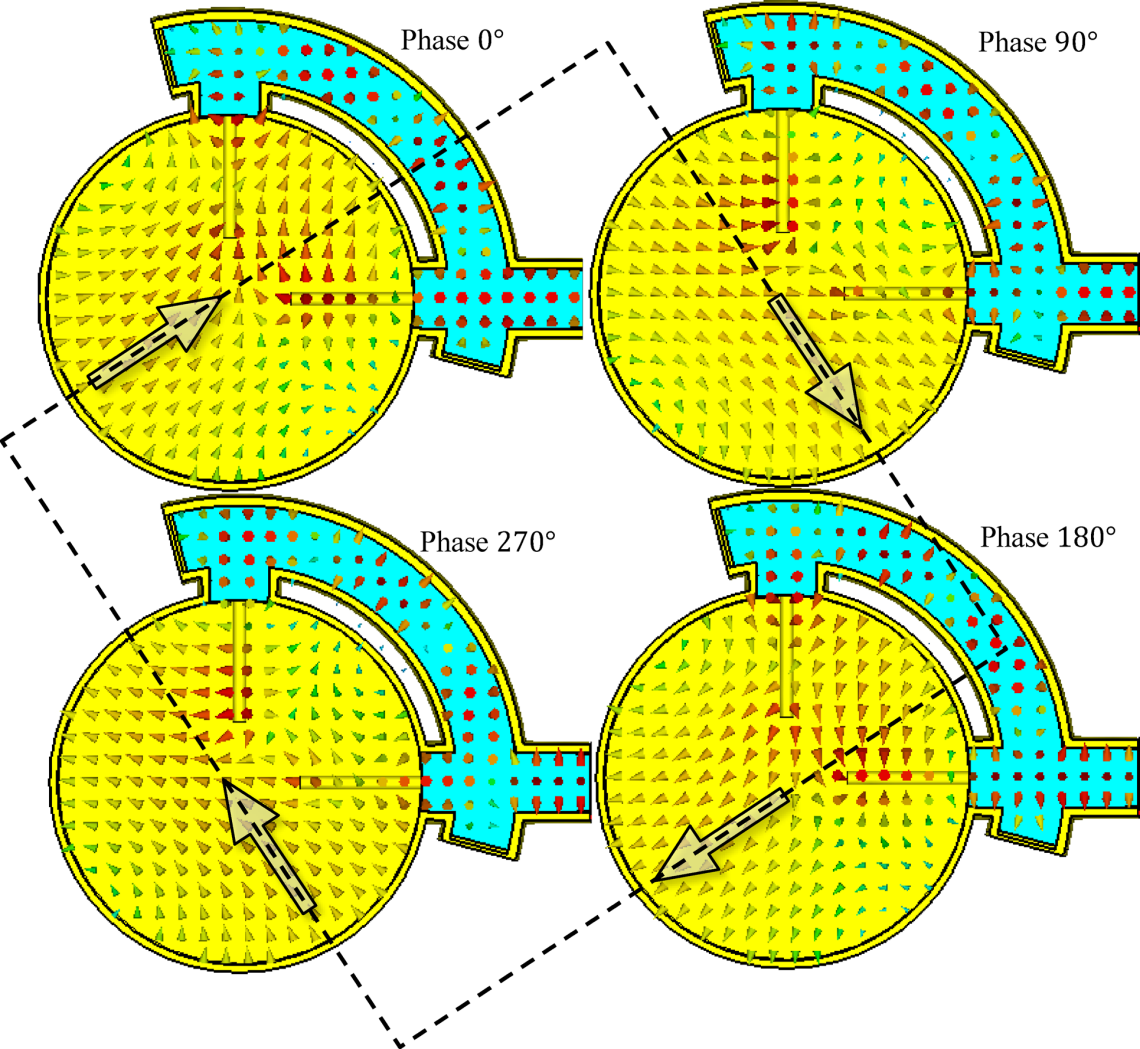
Simulated electric field distributions at sequential phase steps of 0°, 90°, 180°, and 270°, resulting from excitation at port 1 of the antenna at 10 GHz with port 2 short-circuited. The rotation of the electric field vector in right-angle steps demonstrates circular polarization. Observed from the positive z-direction (from the closed end toward the open end), the rotation occurs in a right-handed sense, confirming the generation of RHCP and validating the effective operation of the coaxial line polarizer.

**Fig. 32:**
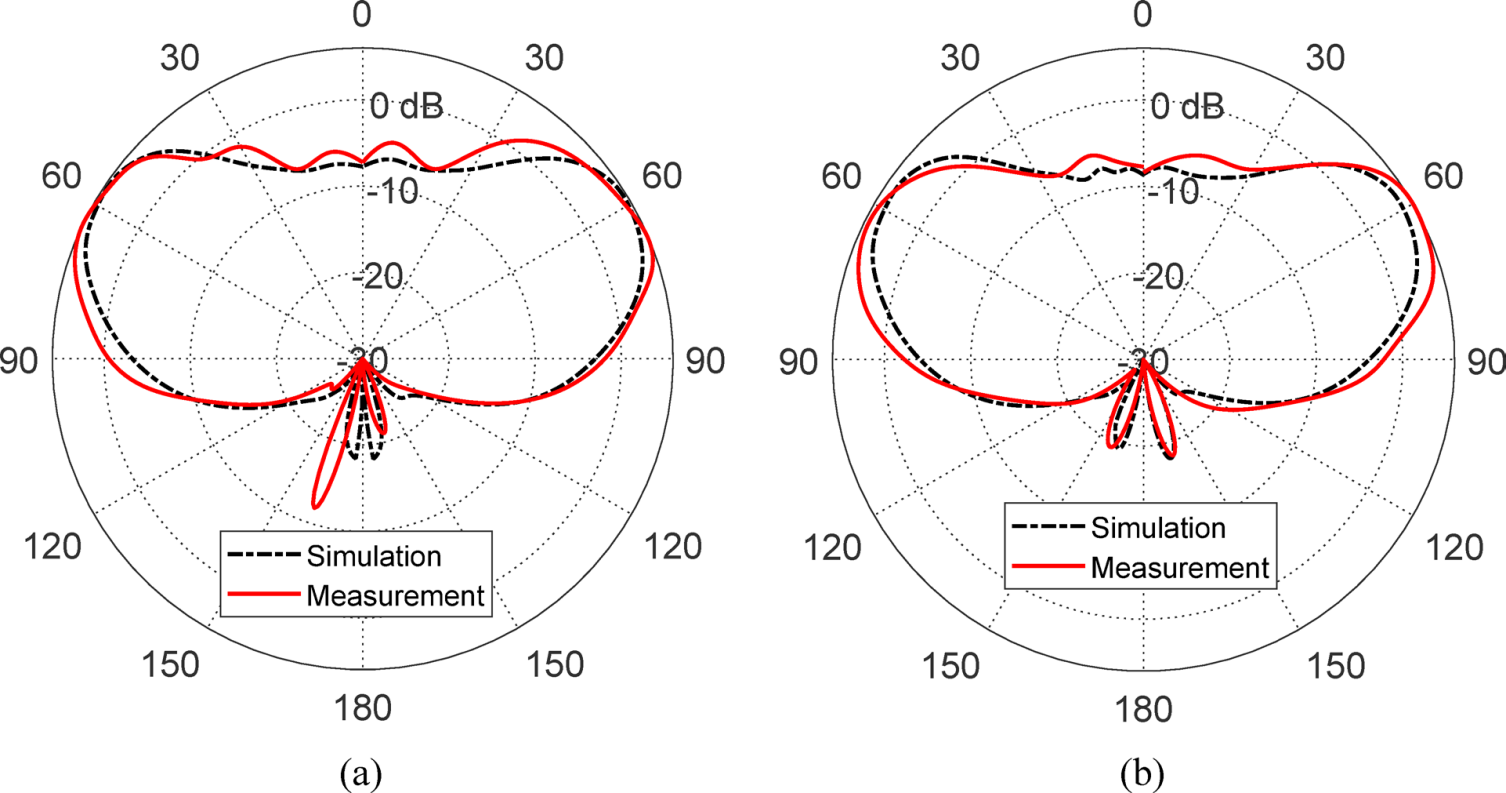
Measured and simulated gain patterns at 10 GHz in the elevation planes: (**a**) $\phi = 0^\circ$ and (**b**) $\phi = 90^\circ$.

This section presents a comprehensive evaluation of the proposed isoflux antenna’s performance, highlighting its key characteristics through Figs. 28, 29, 30, 31 and 32. The analysis includes both simulated and measured results, offering insights into the antenna’s impedance matching, isolation between ports, axial ratio behavior, and radiation patterns. These characteristics are critical for assessing the antenna’s suitability for low LEO satellite communication systems. Particular attention is given to ensuring circular polarization purity, broad impedance bandwidth, and conical radiation suitable for isoflux coverage. The results confirm that the antenna meets the stringent requirements for satellite-ground links, including consistent gain coverage within the visible Earth cone and effective port decoupling for dual-polarized operation.

#### Reflection coefficient at the feeding port

Figure 29 illustrates the frequency response of the reflection coefficient magnitude $(\left| {S_{11} } \right|$) at port 1 of the proposed antenna, intended for RHCP. Both the measured and simulated results show a consistent impedance matching band ranging from 8.75 GHz to 11.25 GHz, within which $\left| {S_{11} } \right|$ remains below the ${-}10{\text{ dB}}$ threshold. This indicates good impedance matching and minimal reflection in this frequency range. The close agreement between the measured and simulated curves validates the accuracy of the antenna design and fabrication. Minor deviations between the two results may be attributed to fabrication tolerances, measurement uncertainties, and slight differences in material properties or boundary conditions not fully captured in simulation.

#### Ports Isolation

Figure 30 illustrates the isolation performance between the two antenna ports as indicated by the transmission coefficient $\left| {S_{21} } \right|$, derived from both measurement and simulation. Across the impedance matching band from 8.75 to 11.25 GHz, the $\left| {S_{21} } \right|$ values consistently remain between $- 20{\text{ dB}}$ and $- 12{\text{ dB}}$. This indicates a moderate to good level of port isolation, with values closer to $- 20{\text{ dB}}$ reflecting better isolation. The results suggest that the antenna exhibits acceptable mutual coupling behavior over its operational bandwidth, ensuring minimal power leakage between the ports. This level of isolation is sufficient for many dual-port applications, such as circularly polarized MIMO or duplex communication systems, where reduced inter-port interference is critical for maintaining signal integrity. The close agreement between simulated and measured curves further confirms the validity of the antenna design and fabrication.

#### Near field distribution

Figure 31 presents the simulated electric field distributions at sequential phase steps of 0°, 90°, 180°, and 270°, resulting from excitation at port 1 of the antenna at 10 GHz, with port 2 short-circuited. These snapshots illustrate how the electric field vector undergoes a spatial rotation in the plane perpendicular to the propagation direction, advancing in 90° increments. This consistent rotation with increasing phase confirms the generation of circular polarization.

Observing the field progression from the viewpoint looking in the positive z-direction (i.e., from the closed end toward the open end of the antenna), it is evident that the electric field vector rotates in a right-handed sense. This indicates that the radiated wave is RHCP. The results clearly demonstrate that the coaxial line polarizer functions as intended, providing the required phase shift and amplitude balance to generate RHCP radiation with high fidelity.

#### Gain patterns

Figure 32 presents the simulated and measured gain patterns of the antenna at $10{\text{ GHz}}$ in the elevation planes $\phi = 0^\circ$ and $\phi = 90^\circ$. The patterns exhibit a characteristic conical shape for the RHCP radiation, which is essential for isoflux coverage in LEO satellite applications. The maximum gain of approximately $6{\text{ dBic}}$ is achieved at an elevation angle of about $63^\circ$ off the nadir, while the minimum gain reaches ${-}6{\text{ dBic}}$ near the boresight direction. This conical beam profile ensures a relatively uniform power density over the Earth’s surface when viewed from a satellite at a $700{\text{ km}}$ altitude, thereby meeting the isoflux requirement. In particular, the antenna maintains sufficient gain for ground stations located at elevation angles as low as $10^\circ$, which is typically the minimum required to maintain reliable communication links. The close alignment between simulation and measurement results in both principal planes confirms the antenna’s capability to deliver the desired radiation characteristics and satisfy the performance criteria for LEO satellite communication systems.

The achieved antenna gain of 6 dBic is well-suited for LEO satellite missions, where the communication link distance is relatively short and design priorities emphasize wide beam coverage or isoflux radiation patterns rather than high directivity. In such scenarios—particularly for non-geostationary small satellite platforms supporting telemetry, tracking, and command (TT&C) operations, as well as payload data transmission (e.g., downlinking Earth observation images to ground stations)—a gain in the range of 6–8 dBic is commonly employed. This level of gain provides an effective balance between coverage area, polarization performance, and size and weight constraints, which are critical for compact spaceborne systems. Moreover, the proposed antenna is specifically designed to provide circular polarization and a tailored radiation profile, both of which are essential in satellite communication environments where the relative orientation between the satellite and the ground station changes continuously during orbital passes.

#### Axial ratio

**Fig. 33:**
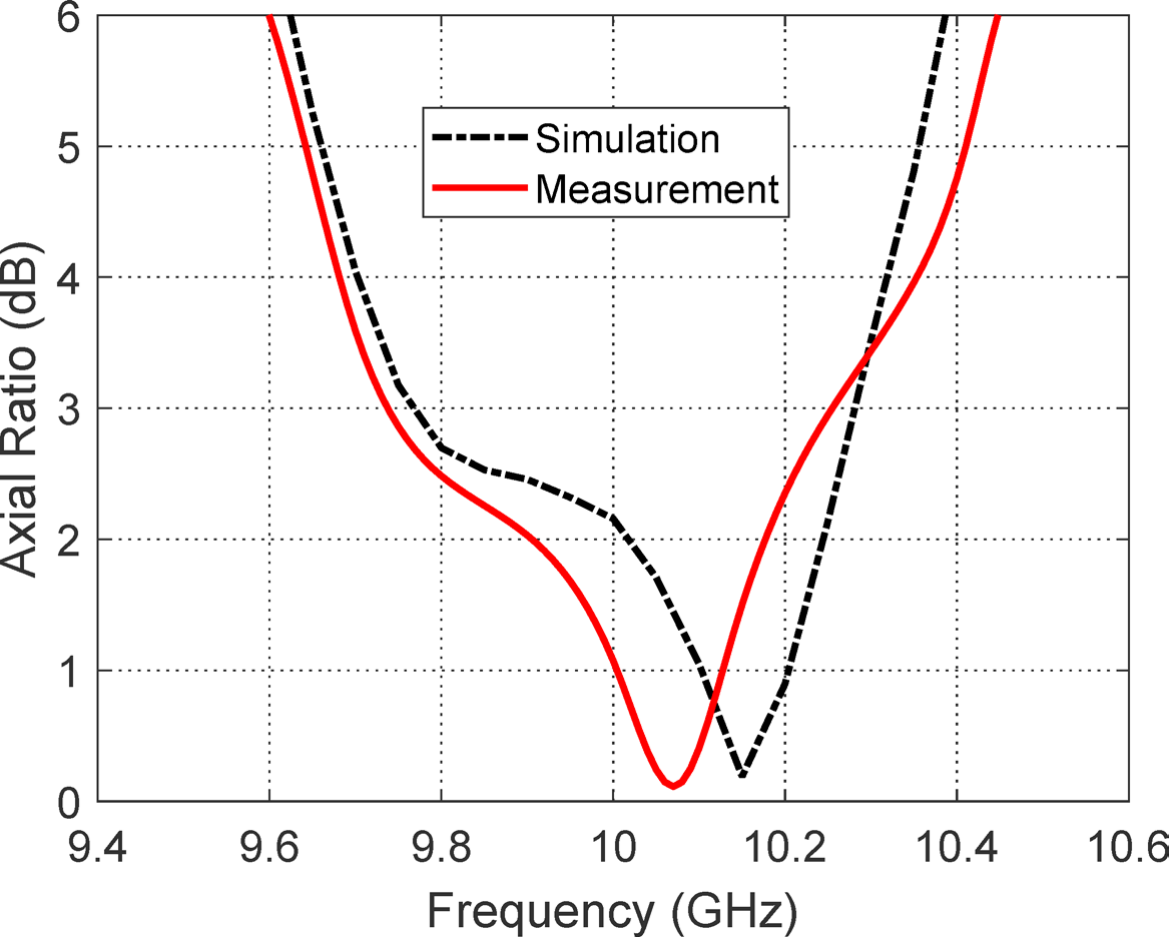
Simulated and measured frequency response of the axial ratio when the antenna is excited through port 1.

Figure 33 shows the frequency response of the axial ratio when the antenna is excited through port 1, comparing simulated and measured results. The axial ratio remains below 3 dB within the CP bandwidth of 9.75–10.25 GHz, indicating a well-defined CP operation over this range. The 6 dB axial ratio bandwidth is broader, extending from 9.6 to 10.4 GHz, reflecting the frequency span where the polarization remains moderately elliptical and acceptable for certain CP applications. Notably, the minimum axial ratio is 0.15 dB, occurring at $10.15{\text{ GHz}}$, which highlights excellent circular polarization purity at the design frequency. The agreement between simulation and measurement confirms the effectiveness of the antenna design in achieving stable and high-quality CP performance around the intended frequency band.

#### Cross plarized components

**Fig. 34:**
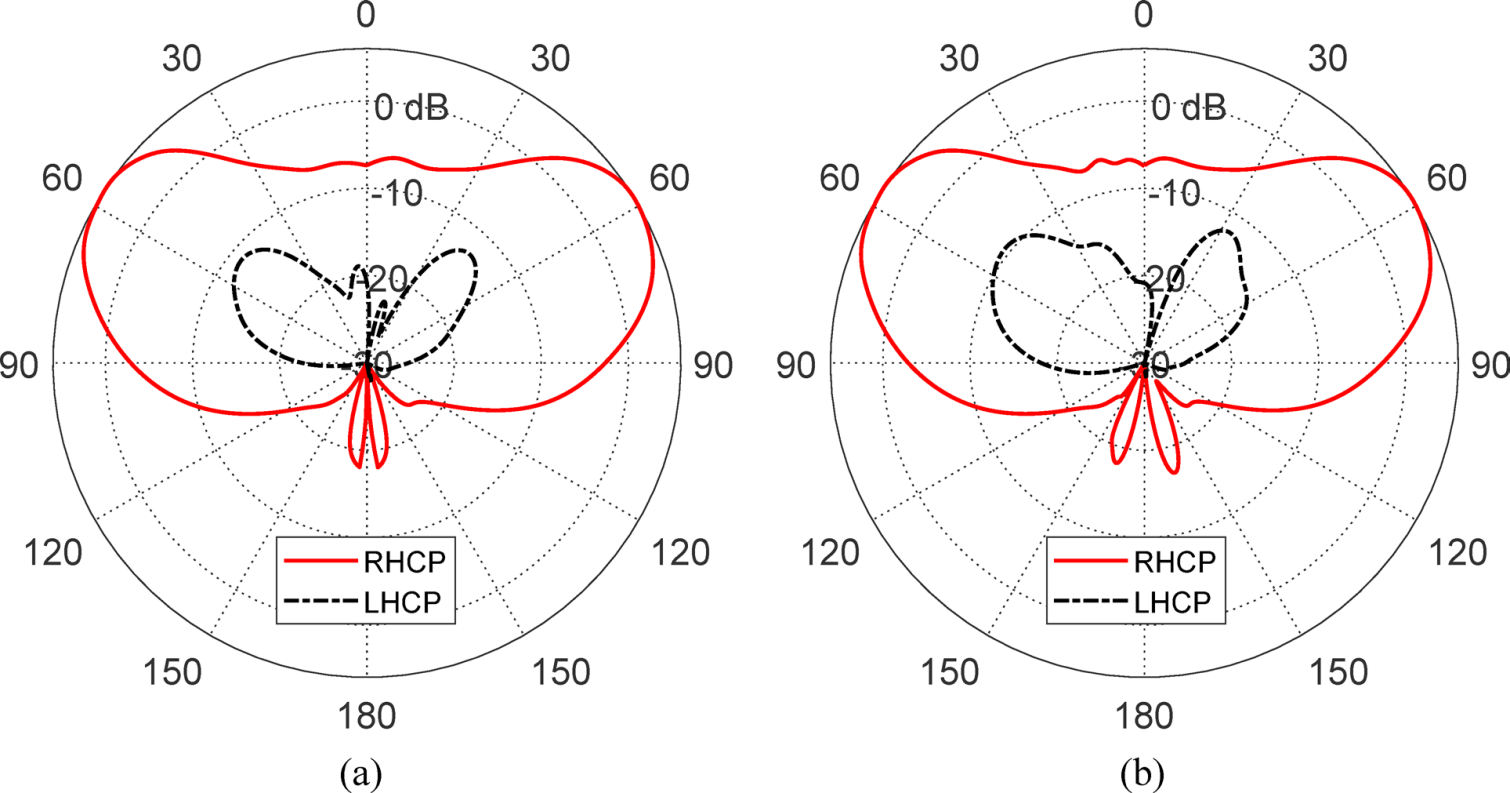
Copolarized (RHCP) and cross-polarized (LHCP) gain patterns at 10 GHz when the antenna is fed through port 1, shown in the elevation planes: (**a**) $\phi = 0^\circ$ and (**b**) $\phi = 90^\circ$.

Figure 34 illustrates the copolarized (i.e., RHCP) and cross-polarized (i.e., LHCP) gain patterns at 10 GHz when the antenna is excited through port 1, evaluated in the elevation planes $\phi = 0^\circ$ and $\phi = 90^\circ$. The radiation is clearly dominated by the RHCP, as intended, with the copolarized RHCP component significantly exceeding the cross-polarized LHCP component across most directions. The ellipticity ratio ($E_{R} /E_{L}$) exceeds $16{\text{ dB}}$ in the majority of the angular region, confirming strong circular polarization purity and excellent isolation between the orthogonal polarizations. This performance is indicative of a well-designed circularly polarized antenna and verifies the antenna’s effectiveness in minimizing undesired polarization components during operation.

#### Antenna efficinecy

**Fig. 35:**
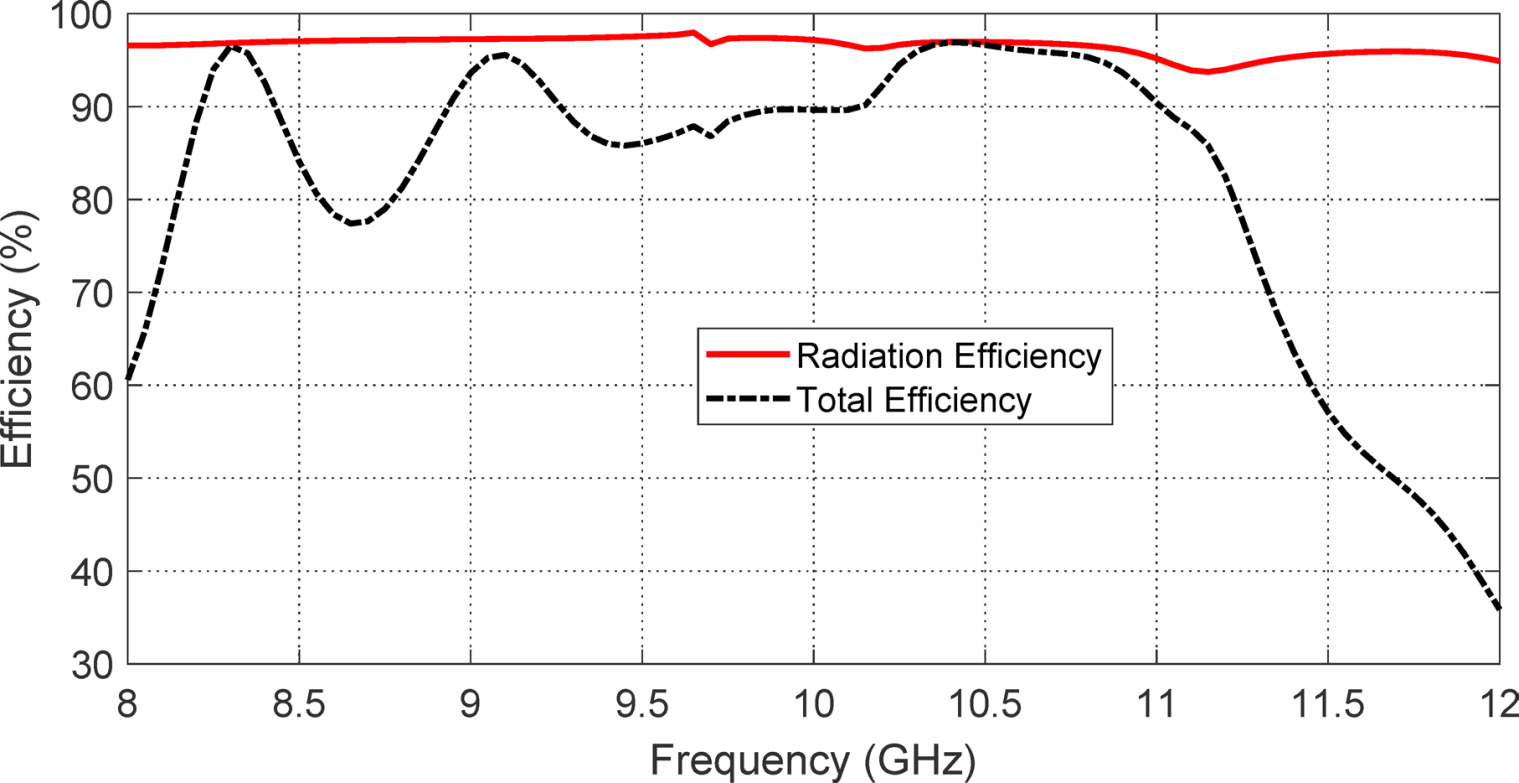
Frequency dependence of the radiation and total efficiencies of the proposed PANI backed lens antenna fed with open-ended circular waveguide on the frequency over a wide frequency range.

The frequency dependencies of the radiation and total efficiencies of the proposed PANI backed lens antenna fed with open-ended circular waveguide on the frequency over a wide frequency range are presented in Fig. 35. The radiation efficiency is almost constant and equal to 98% over the entire frequency range of investigation (8–12 GHz). The total efficiency is maintained at almost 90% over the operational frequency band (9.75–10.25 GHz). Such high efficiency is achieved in spite of the presense of the PANI absorbing disc.

The observed near-perfect absorption efficiency of the proposed antenna structure can be directly attributed to the unique electromagnetic characteristics of the PANI composite material used as a lossy absorber. Polyaniline, a conducting polymer, exhibits frequency-dependent complex permittivity and moderate conductivity, making it highly effective in dissipating incident electromagnetic energy over a targeted frequency band.

At the microstructural level, the PANI material features a porous, interconnected morphology, as confirmed by SEM analysis. This irregular structure promotes multiple internal reflections and extended interaction paths for the incident waves, thereby enhancing energy dissipation through dielectric loss mechanisms. Furthermore, the nano-scale conductive domains embedded within the PANI matrix contribute to additional Ohmic losses, which convert incoming microwave energy into heat.

From an electromagnetic perspective, the absorber’s impedance is closely matched to that of free space, minimizing reflection at the interface and allowing efficient energy transfer into the material. Once inside, the combination of optimized dielectric loss tangent and moderate conductivity ensures that the energy is effectively attenuated, rather than re-radiated or reflected. This impedance matching and absorption synergy is particularly well-tuned at the operational frequency of 10 GHz, where the material exhibits its peak absorption response.

Overall, the near-unity absorption efficiency demonstrated by the proposed antenna system reflects not only the appropriate integration of the PANI absorber but also the careful tuning of its physical and electrical properties to achieve optimal performance. This highlights the importance of material engineering at both the macro and micro scales in the design of high-performance electromagnetic absorbers for satellite communication systems.

## Comparison with related works

This section prvides comparison along with a proposed reference table to clearly demonstrate the novelty and relevance of the proposed isoflux antenna compared to recent work in the field. Table 2 prsents a comparison of the proposed antenna with recently reported circularly polarized antenna designs targeting satellite applications. Key performance metrics such as polarization technique, frequency band, gain, axial ratio, and antenna efficiency, and intended application are compared. The table highlights the novelty of the proposed design in achieving isoflux radiation with circular polarization and moderate gain, using a compact coaxial polarizer structure suitable for LEO satellite systems.

**Table 2:** Comparison of the proposed antenna with recently reported circularly polarized antenna designs targeting satellite applications.

References	Antenna type	Polarizer type	Frequency band (GHz)	Axial ratio (dB)	Gain (dBic)	Advantages of the Proposed work
^27^	Quadrifilar Helix	Inherent	2–2.2 GHz	5	3	Higher gain with isoflux profile, better axial ratio and dual polarized
^28^	Waveguide slot array	Waveguide-based CP	Dual-band at 12 GHz for Tx and at 14.5 GHz for Rx	3 dB for Tx band 4 dB for Rx band	3	Better axial ratio, and higher gain with isoflux profile
^29^	Waveguide with diaphragms	Waveguide polarizer	10.7–12.8	1.5	N/A	Better axial ratio, simpler two-port coaxial polarizer
^30^	Coaxial-to-waveguide converter Antenna	Mode converter antenna	9.95–10.05	1.43	Low gain $\sim 0$	Wider frequency band, better axial ratio, higher gain with isoflux profile, and simpler coaxial polarizer
^31^	Patch + superstrate + cavity-backed	Sequential phase + cavity	7.6–9.7	≤ 3	9.3	More compact design, higher gain, but lacks absorber-enhanced isoflux shaping
^32^	Dielectric lens fed by phased array	Circular polarization via array	26–30	≤ 1.5	17.5	Excellent scanning and gain, but larger size and higher complexity
^33^	Spherical Luneburg lens with printed feed	Inherent via lens symmetry	8.2–12.5	≤ 3	11.3	High gain and broad bandwidth, but lacks polarization filtering control
Present	Lens backed by PANI absorber	Two-port coaxial polarizer	9.75–10.25	0.05	6	

Table 2 provides a comprehensive comparison between the proposed isoflux circularly polarized antenna and recent state-of-the-art antenna designs targeting satellite applications^27–30^, as well as newly added high-performance structures^31–33^. The proposed design demonstrates a unique combination of technical strengths and practical advantages relevant to LEO satellite communications.

(i) Compact coaxial polarizer with dual-port flexibility:
  Compared to the waveguide-based polarizers in^28^ and diaphragm-loaded waveguides in^29^, the proposed antenna adopts a simple and compact coaxial quarter-wave polarizer that supports dual-port excitation. This not only reduces the volume and complexity of the feed network but also enables selectable RHCP/LHCP operation via phase control, offering significant operational flexibility, unlike the inherent (but fixed) polarization in^27^ or^31^.
(ii) Isoflux radiation pattern for Earth coverage:
  Most high-gain circularly polarized designs in^31,33^ focus on directive beams, often optimized for point-to-point or narrow coverage. In contrast, the proposed antenna is tailored for producing a uniform isoflux pattern, ensuring consistent power density over the Earth’s surface, essential for satellite-to-ground links without active beam steering or complex lens shaping.
(iii) Balanced gain and coverage performance:
  While antennas in^28–30^ deliver moderate to low gains (0–3 dBic), and^27^ exhibits limited efficiency, the proposed design achieves approximately 6 dBic gain while preserving a broad radiation profile. This positions it between high-gain directive antennas^31,33^ and low-gain omnidirectional systems^28^, thus addressing both link budget and wide-area coverage in LEO missions.
(iii) Enhanced axial ratio performance
  The axial ratio of the proposed design is significantly improved (≈ 0.05 dB), surpassing the values reported in^27–30^ and^31–33^, which typically range from 1.0 to 5 dB. This indicates superior polarization purity, contributing to reduced polarization mismatch losses in satellite links and higher communication reliability.
(iv) Material and manufacturing innovation:
  In contrast to the spherical Luneburg lens approach in^33^ or GRIN lens fabrication in^32^, the proposed antenna leverages a planar PANI-based absorber backed by a dielectric lens. This structure simplifies manufacturing, reduces weight, and enables tailored electromagnetic absorption to suppress back radiation, enhancing the front-to-back ratio and antenna efficiency without sacrificing compactness.

In summary, the proposed antenna distinguishes itself through a synergistic integration of coaxial polarizer simplicity, isoflux pattern shaping, enhanced polarization purity, and compact fabrication, making it exceptionally well-suited for modern LEO satellite missions demanding efficient and adaptable antenna systems.

## Conclusion

This paper has presented the design, development, and optimization of a high-performance isoflux circularly polarized antenna tailored for X-band fully-duplex communication in LEO satellite systems. The antenna employs an open-ended circular waveguide excited by a dual-port polarizer, enhanced with a concentric dielectric lens and an external reflector disc to generate a conical beam covering a wide angular sector of approximately $126^\circ \times 126^\circ$, ensuring ground coverage from $\pm 63^\circ$ off-nadir. To further improve circular polarization purity, suppress back radiation, and enhance impedance matching, a nano-material-based absorbing disc composed of PANI is integrated between the lens and the reflector. Through extensive parametric studies and electromagnetic simulations, the antenna achieves a 3-dB axial ratio bandwidth of $500{\text{ MHz}}$ ($9.75{-}10.25{\text{ GHz}}$) and an impedance matching band spanning $8.75{-}11.25{\text{ GHz}}$. The realized gain reaches $6.0{\text{ dBic}}$ at the targeted radiation angles, with polarization isolation and radiation characteristics well-suited for high-reliability satellite communications. The incorporation of functional nano-materials, validated through XRD and SEM analysis, highlights a promising pathway for performance enhancement in advanced spaceborne antenna systems.

The main contributions of this work are: (i) A dual-port circular polarization scheme using a broadband orthomode transducer (OMT) with > 20 dB isolation across X-band, enabling efficient LHCP transmission and RHCP reception for full-duplex LEO terminals; (ii) A top-cut dielectric lens producing a conical beam with ± 63° coverage from nadir; (iii) Novel use of a DBSA-doped polyaniline (PANI) nano-material absorber integrated between the lens and reflector—distinct from prior external or backing absorbers; and (iv) Improved radiation performance through this internal PANI absorber placement, yielding sidelobe suppression, surface wave reduction, and enhanced axial ratio bandwidth without increasing antenna size.

## Data Availability

The datasets used and/or analysed during the current study are available from the corresponding author (H. abuklill) on reasonable request.
